# ClustAll: An R package for patient stratification in complex diseases

**DOI:** 10.1371/journal.pcbi.1012656

**Published:** 2024-12-13

**Authors:** Asier Ortega-Legarreta, Sara Palomino-Echeverria, Estefania Huergo, Vincenzo Lagani, Narsis A. Kiani, Pierre-Emmanuel Rautou, Nuria Planell Picola, Jesper Tegner, David Gomez-Cabrero

**Affiliations:** 1 Unit of Translational Bioinformatics, Navarrabiomed—Fundación Miguel Servet, Universidad Publica de Navarra (UPNA), IdiSNA, Pamplona, Spain; 2 Biological and Environmental Science and Engineering Division, King Abdullah University of Science and Technology (KAUST), Thuwal, Saudi Arabia; 3 SDAIA-KAUST Center of Excellence in Data Science and Artificial Intelligence, Thuwal, Saudi Arabia; 4 Institute of Chemical Biology, Ilia State University, Tbilisi, Georgia; 5 Algorithmic Dynamics lab, Karolinska Institutet, Solna, Sweden; 6 Université Paris-Cité, Inserm, Centre de recherche sur l’inflammation, UMR, Paris, France; 7 AP-HP, Hôpital Beaujon, Service d’Hépatologie, DMU DIGEST, Centre de Référence des Maladies Vasculaires du Foie, FILFOIE, ERN RARE-LIVER, Clichy, France; 8 Computational Biology Program, Universidad de Navarra, CIMA, Instituto de Investigación Sanitaria de Navarra (IdiSNA), Navarra, Spain; 9 Unit of Computational Medicine, Department of Medicine, Center for Molecular Medicine, Karolinska Institutet, Karolinska University Hospital, Stockholm, Sweden; 10 Computer, Electrical and Mathematical Sciences and Engineering Division, King Abdullah University of Science and Technology (KAUST), Thuwal, Saudi Arabia; US Army Medical Research and Materiel Command: US Army Medical Research and Development Command, UNITED STATES OF AMERICA

## Abstract

In the era of precision medicine, it is necessary to understand heterogeneity among patients with complex diseases to improve personalized prevention and management strategies. Here, we introduce ClustAll, a Bioconductor package designed for unsupervised patient stratification using clinical data. ClustAll is based on the previously validated methodology ClustAll, a clustering framework that effectively handles intricacies in clinical data, including mixed data types, missing values, and collinearity. Additionally, ClustAll stands out in its ability to identify multiple patient stratifications within the same population while ensuring their robustness. The updated implementation of ClustAll features S4 classes, parallel computing for enhanced computational efficiency, and user-friendly tools for exploring and comparing stratifications against clinical phenotypes. The performance of ClustAll has been validated using two public clinical datasets, confirming its effectiveness in patient stratification and highlighting its potential impact on clinical management. In summary, ClustAll is a powerful tool for patient stratification in personalized medicine.

## 1 Introduction

Stratification emerges as a necessary approach in precision medicine, especially in complex diseases, where identifying subgroups of patients can enhance tailored prevention and management strategies. Many existing clinical classifications rely solely on scoring systems designed to predict patient outcomes, potentially overlooking essential features that can introduce diversity in patient populations and may directly impact prognosis [1]. In other words, a similar outcome does not imply a similar pathophysiology and/or disease type. Therefore, unsupervised patient stratification is necessary for developing precision medicine strategies for complex diseases. Despite the availability of tools that offer a variety of clustering algorithms, existing pipelines lack a generic framework capable of effectively managing common complexities in clinical data, such as mixed data types, missing values, and collinearity [2–4].

ClustALL, a validated stratification framework, was developed to address the previously mentioned limitations and accounts for two additional features [5]. First, it allows the identification of *multiple stratifications* within the same population. Second, it integrates two different *robustness* criteria to identify reliable stratification solutions. These criteria include **(I)** population-based robustness–an evaluation of stratification stability through bootstrapping–and **(II)** parameter-based robustness–which assesses the stability of the stratification under varied parameter alterations, such as dissimilarity metric or clustering method. While the original ClustALL implementation successfully stratified patients with acute decompensation of cirrhosis [5], it lacked flexibility, required extensive computational time, and did not allow user-friendly exploration of the results.

To address these gaps and provide a valuable stratification tool for the community, we expanded the original ClustALL algorithm into an *R pipeline* named ClustAll [6,7]. The enhanced implementation incorporates an *S4-oriented design* to ensure stability and efficiency for complex data analysis, consistent behavior, and easy integration into broader workflows [6]. Additionally, we incorporated *parallel computing* to reduce computational times [8,9], added *new functionalities* to investigate and compare stratifications against known labels or between multiple stratifications, and allowed the customization of various *hyper-parameter* values. Lastly, the package includes a *vignette* that explains the step-by-step process using two public clinical datasets.

## 2 Design and implementation

### 2.1 Overview

ClustAll is developed using S4 object-oriented programming and requires R (> = 4.2) [6]. The input data, a data frame or matrix with clinical data, is stored in the ‘ClustAllObject’ object using the ‘createClustAll’ function. A minimum of two features are required as input. ClustAll can handle binary, categorical, and numerical variables. It internally transforms categorical features using a one-hot encoder [10]. Depending on the completeness of the input data, the ClustAll pipeline handles three different scenarios: **(I)** initial complete data (without missing values), **(II)** initial incomplete data (with missing values) imputed within ClustAll, and **(III)** data with missing values that have been imputed externally. See sections 2.2 and 2.3 for more details.

### 2.2 Stratification Workflow considering complete input data (scenario I)

**Fig 1:**
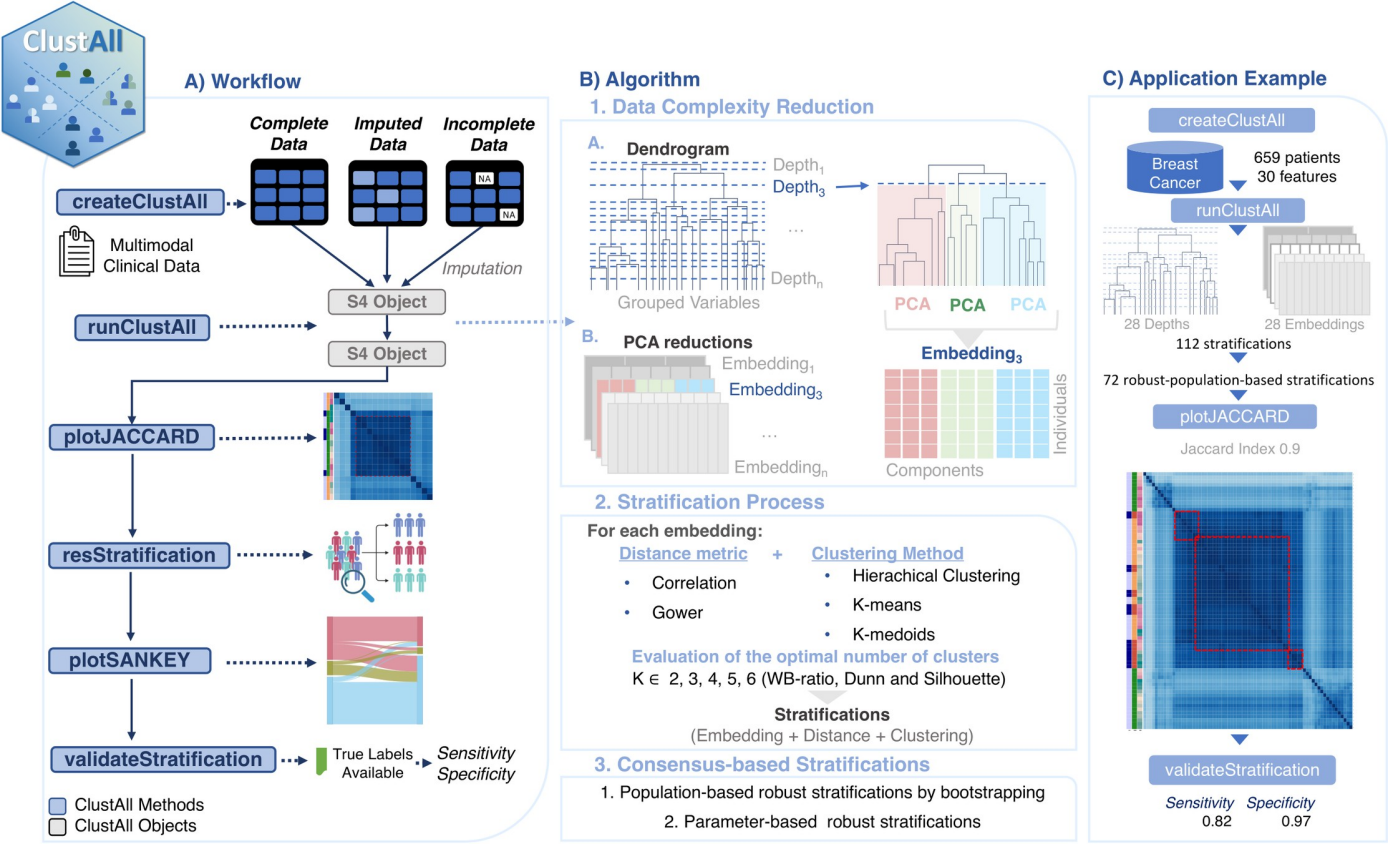
Schematic representation of the ClustAll pipeline. **(A)** Stepwise and main features included in the ClustAll package, illustrating the workflow integrated into the tool for clustering, data analysis, and results visualization; **(B)** Overview of the ClustALL Algorithm Methodology; **(C)** Application example overview. Figure based on Palomino-Echeverria et al. 2024 [5].

After generating the ’ClustAllObject’ object using the ’createClustAll’ function, the ’runClustAll’ method executes the ClustALL algorithm. The ClustALL framework involves three main steps:

- In the first step, Data Complexity Reduction (DCR), multiple data embeddings are created to replace a highly correlated set of variables with lower-dimension projections derived from Principal Component Analysis (PCA). This process explores all relevant groupings derived from a hierarchical clustering-based dendrogram [11]. Consequently, DCR computes an embedding for each depth in the dendrogram (see **Fig 1B**, Step 1).
- In the second step, the Stratification Process (SP), ClustALL calculates and preliminarily evaluates stratifications for each embedding by computing a stratification for each feasible combination of embedding, dissimilarity metric, and clustering method, considering a predefined range of cluster numbers (default is 2 to 6). The optimal number of clusters is determined using three internal validation measures: the sum-of-squares (WB-ratio), Dunn index, and average Silhouette width (see Fig 1B, Step 2) [12,13]. Each combination yields a stratification comprising ’embedding + distance metric + clustering method’.
- The final step, Consensus-based Stratifications (CbS), non-robust stratifications are first filtered out using bootstrapping [14]; as a result, stratifications with a stability below 85% are excluded. From the remaining robust stratifications, representative outcomes are selected based on similarity. This process, known as parameter-based robustness, involves "clustering of stratifications" using the Jaccard index [15] as the distance metric. The results are visualized in a heatmap, which aids in identifying groups of similar stratifications (see **Fig 1B**, Step 3). For each group, one stratification is selected as the representative. As a result, the ClustALL algorithm and, consequently, ’runClustAll’ may generate none, one, or multiple robust alternatives for stratifying the population. These stratifications are denoted by the embedding number and a letter corresponding to the combination of dissimilarity metric and clustering method (**Table 1**).

**Table 1:** ClustALL methods nomenclature.

Nomenclature	Distance Metric	Clustering Method
a	Correlation	K-means
b	Correlation	Hierarchical clustering
c	Gower	K-medoids
d	Gower	Hierarchical clustering

### 2.3 Stratification workflow considering input data with missing values (scenarios II and III)

ClustALL can handle missing data. If the input data contains missing values, ClustAll can either impute the data within the ’createClustAll’ function, which directly applies the ‘*mice’* function from the mice package (scenario II), or accept a mids object (Multiple Imputed DataSet) generated externally also with mice (scenario III) [16]. A mids object is a specialized data structure that includes both the original incomplete dataset, its multiple imputed versions, and the parameters used to generate them. Note that multiple imputations can be performed to avoid potential bias introduced by relying on a single imputation. For each imputation, the workflow undergoes the following modifications:

- First, a dendrogram and its associated depths are computed based on the original dataset containing missing values. The DCR step is implemented for each calculated depth on the imputed dataset.
- Next, the SP step is applied for each combination of depth, distance metric, and clustering algorithm derived from the imputed dataset. The optimal number of clusters is determined based on consensus from cluster internal validation and the mode of the imputed datasets corresponding to each embedding.
- Subsequently, a distance matrix between individuals is generated by assessing how often two individuals are assigned to the same cluster in each imputation. This matrix then computes a final stratification score using correlation-based distance and hierarchical clustering. The process then proceeds to the CbS step, as previously described.

### 2.4 Interpretation workflow

**Fig 2:**
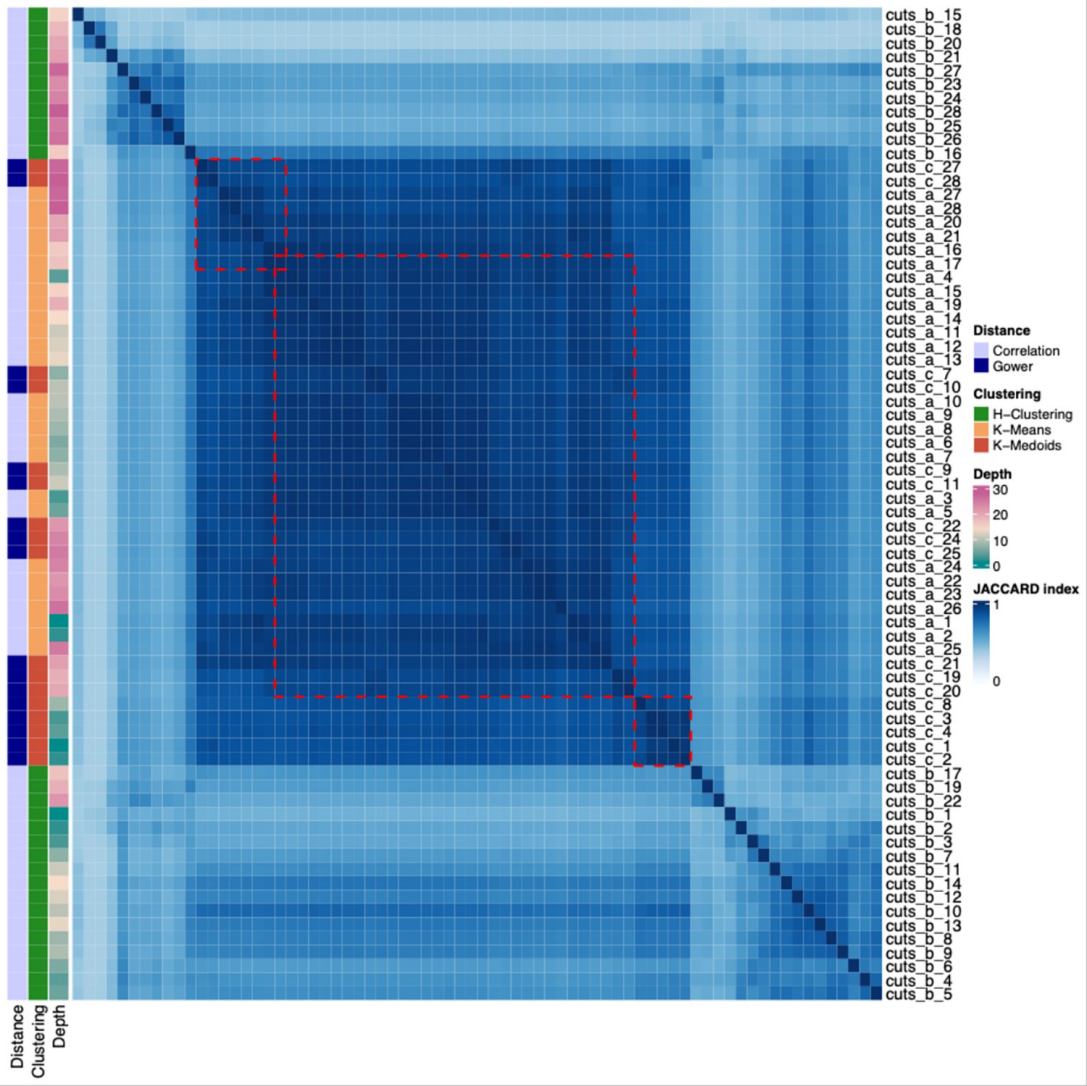
Heatmap with the Jaccard indexes for population-robust stratifications. Heatmap representing the similarity between stratifications using the Jaccard Index. It groups similar stratifications, allowing for the identification of patterns that exhibit similar behavior. The X-axis and Y-axis represent the different stratifications. The color gradient, ranging from blue to white, indicates the Jaccard index values. A darker blue represents a higher Jaccard index, indicating higher similarity between sets, while a lighter blue (approaching white) represents a lower Jaccard index, indicating less similarity. Red dashed lines highlight groups of stratifications that show a high degree of similarity based on the Jaccard index threshold (in this case Jaccard Index is 0.9). The label “Distance” refers to the type of similarity measure used, such as correlation or Gower distance. The label “Clustering” indicates the clustering method applied, such as hierarchical clustering, k-means, or k-medoids. “Depth” refers to the level of embedding, showing in a color range the depths of the dendrogram. *H-Clustering*: hierarchical clustering.

ClustAll offers various *new functionalities* for exploring and interpreting the results. The ‘plotJaccard’ function, utilizing the *complexHeatmap* package [17], displays the Jaccard distance between robust stratifications through a correlation heatmap [15] (**Figs 1A** and **2**). This heatmap includes annotations with the similarity distance, clustering algorithm, and embedding depth used to compute each given stratification (**Table 1**). Similar stratifications are grouped and marked with a discontinuous red rectangle, with the centroid of each group acting as a representative. Stratifications can be retrieved using the ‘resStratification’ function. ClustAll also provides a function for visually comparing sets of representative stratifications with Sankey diagrams using the *networkD3* package [18]. Additionally, it allows for evaluating the performance of the selected stratifications against known labels, if available, by calculating sensitivity and specificity using the ‘validateStratification’ function (**Fig 1A**). This function calculates the *sensitivity*, also known as the true positive rate, which is defined as the number of true positives (TP) divided by the sum of the TP and false negatives (FN); and the *specificity*, also known as the true negative rate, which is defined as the number of true negatives (TN) divided by the sum of the true TN and false positives FP. The closer the values of sensitivity and specificity are to 1, the better the model’s performance. When comparing stratification results to known labels, the majority class in each cluster is considered the positive class for that cluster. Finally, selected stratifications can be extracted alongside the initial matrix or data frame using the ‘cluster2data’ method.

Furthermore, the ClustAll implementation allows the users to customize various *hyper-parameter* values. When computing the embeddings, it is possible to set the maximum depth for the initial hierarchical clustering (default is the *number of input variables—2*) [5]. Additionally, it permits specifying the number of imputations to be performed if the input dataset contains missing values and is performed within ClustAll [16]. Lastly, the user can establish the Jaccard index threshold to determine when a pair of stratifications should be considered similar in the CbS step (default is 0.7) [19]. Because the optimal threshold will depend on the analyzed data set, we recommend starting with higher values (e.g., 0.9) and gradually decreasing if necessary; see **S1 Text, sections 6.3.4 and 7.5** for examples.

## 3 Results

The original version of the ClustALL algorithm was applied to stratify a population of patients with acutely decompensated cirrhosis [5]. We analyzed two public clinical datasets to validate its implementation as an R-package and demonstrate its practical applications [20,21]. This allowed us to compare the stratification results produced by ClustAll against known labels.

### 3.1 Dataset 1

ClustAll includes a public dataset of breast cancer data derived from a digitized image of a fine needle aspirate of breast mass from 659 patients (**Fig 1C**). This dataset comprises 30 numerical features (10 variables measured three times) includes binary classification labels (’malignant’ or ’benign’) based on clinical diagnosis that serve as ground truth. (**Table 2**) [20]. These labels are removed from the input data used for unsupervised stratification and are only used afterward for validation purposes.

**Table 2:** Breast Cancer Winconsin (Diagnostic) dataset attributes description.

Variable Name	Role	Type	Description
Diagnosis	Target	Categorical	Tumour diagnosis
Radius	Feature	Continuous	Mean distances from the center to points on the perimeter
Texture	Feature	Continuous	Standard deviation of grey-scale values
Perimeter	Feature	Continuous	Perimeter of the breast mass affected by the cancer
Area	Feature	Continuous	Area of the breast mass affected by the cancer
Smoothness	Feature	Continuous	Local variation in radius lengths
Compactness	Feature	Continuous	(Perimeter^2 / Area) - 1.0
Concavity	Feature	Continuous	Severity of concave portions of the contour
Concave points	Feature	Continuous	Number of concave portions of the contour
Symmetry	Feature	Continuous	Degree of symmetry in the shape and structure of the breast mass, with higher values indicating greater symmetry and lower values indicating asymmetry
Fractal dimension	Feature	Continuous	“Coastline approximation” - 1

In this scenario, ClustAll produced 112 distinct stratifications. These groupings were obtained by combining 28 embeddings created from 30 input features. Out of the 112 stratifications, 72 remained consistent after bootstrapping.

In line with the notion of parameter-based robustness, it is feasible to arrive at similar stratifications through different methods. In this sense, we identified three alternatives for grouping the population, specifying a Jaccard index similarity of 0.9. These stratifications are depicted by the discontinuous red squares (**Fig 2**). **(i)** The first stratification option is derived from 8 alternatives, which includes correlation distance with K-means and Gower with K-medoids, along with different embedding depths; **(ii)** the second option is derived from 32 alternatives based on correlation and Gower distances with K-means and K-medoids, along with different embedding depths; **(iii)** the third option comprises 5 instances, derived from correlation distance with K-medoids and different embeddings depths.

The representative stratifications corresponded to "cuts_a_28", "cuts_c_9", and "cuts_c_4". It is worth noting that when these three stratifications were assessed for sensitivity and specificity against known labels, the results showed values higher than 80% and 90%, respectively (**Table 3**). The consistency across different methodological approaches demonstrates both the statistical robustness and methodological reliability of these stratifications.

**Table 3:** *Sensitivity and specificity for the stratification representatives*. The performance metrics for the representative stratifications identified by ClustAll when applied to the breast cancer dataset. The "Nomenclature" column shows the identifier for each stratification. "Distance Metric" and "Clustering Method" columns indicate the similarity measure and clustering algorithm used, respectively. "Embedding depth" refers to the level in the dendrogram at which the embedding was created during the Data Complexity Reduction step. "Sensitivity" shows the proportion of true positive cases (malignant tumors) correctly classified by the stratification. "Specificity" indicates the proportion of true negative cases (benign tumors) correctly classified. Both are calculated by comparing the stratification results to the reference column (*true labels*) in the dataset.

Nomenclature	Distance Metric	Clustering Method	Embedding depth	Sensitivity	Specificity
**cuts_a_28**	**Correlation**	**K-means**	**28**	**0.8208**	**0.9748**
**cuts_c_9**	**Gower**	**k-medoids**	**9**	**0.8585**	**0.9580**
**cuts_c_4**	**Gower**	**k-medoids**	**4**	**0.8821**	**0.9664**

To demonstrate ClustAll’s ability to handle missing data, we included a modification of the breast cancer dataset with random missing values. For this purpose, we generated a random number to determine the number of columns that would contain missing values. Then, we generated random percentages to specify the proportion of missing values in each of those columns. Missing values were inserted entirely at random (MCAR) [22]. After applying ClustAll (scenarios II and III) and specifying a Jaccard index stratification similarity threshold of 0.9, we obtained one stratification comprised of 16 alternatives (Fig D in S1 Text). These stratifications included correlation distance with K-means, Gower with K-medoids, and various embeddings. The representative (centroid) corresponded to “cuts_a_2” and had a sensitivity of 0.8443 and specificity of 0.9580.

### 3.2 Dataset 2

We analyzed a second public dataset initially designed to develop predictors of heart failure based on clinical test data [21]. From now we will refer to it as the heart attack dataset. This dataset consists of 918 patient records and includes 11 clinical features, comprising 6 categorical and 5 numerical, with no missing values. This dataset includes the clinical outcome represented by the variable ’HeartDisease’ (0 = No, 1 = Yes), which serves as the ground truth. Like the breast cancer dataset, these clinical diagnosis labels are excluded from the input data and used solely for validation purposes. In this instance, specifying a Jaccard index similarity of 0.9 ClustAll retrieved one stratification option, comprised of 5 alternatives, in which centroid “cuts_a_9” achieved a specificity and specificity rates of 0.8740 and 0.8244, respectively (**Fig F in S1 Text**).

More detailed analyses and results for both public datasets are provided in the S1 Text.

### 3.3 ClustAll vs other methods

We conducted a benchmarking study to evaluate ClustAll’s performance relative to other approaches in scenarios where the labels were known but not disclosed during the stratification. We compared the results of ClustAll with those of standard clustering algorithms by running the analysis multiple times using bootstrapping techniques (**Table 4**). We assessed **(i)** the complete breast cancer dataset bootstrapping 100 times, **(ii)** the breast cancer dataset with missing values and imputed 10 times and bootstrapping 10 times, and **(iii)** the heart attack dataset bootstrapping 100 times [20,21]. The results were evaluated by calculating the average sensitivity and specificity across the bootstrap samples, using the known labels as a reference. We also assessed the stability of the clusters by calculating the average similarity to the originally defined clusters. It is important to note that ClustAll automatically determines the optimal number of clusters, so limiting the cluster number beforehand is not required. For the other algorithms, we set the number of clusters to 2.

**Table 4:** ClustAll performance against standard clustering algorithms. A comparative analysis of ClustAll’s performance against standard clustering algorithms across multiple datasets. The "Method" column lists the clustering approaches evaluated, including ClustAll and various combinations of distance metrics and clustering algorithms. "Sensitivity" and "Specificity" columns show the average accuracy of each method in identifying positive and negative cases, respectively, when compared to the known reference column (*true labels*). These values are calculated across multiple bootstrap samples to ensure reliability. The "Stability" column indicates the consistency of cluster assignments across different bootstrap iterations, with higher values suggesting more robust clustering. Results are provided for three scenarios: the complete breast cancer dataset, the breast cancer dataset with imputed missing values, and the heart attack dataset.

Method	Sensitivity	Specificity	Stability
**Breast cancer (complete dataset)**			
**ClustAll**	0.9172113	0.8487858	87.59379
**Correlation +** **K-means**	0.9970835	0.6075585	99.46427
**Correlation +** **Hierarchical clustering**	0.2801391	0.6599774	58.79239
**Gower +** **K-medoids**	0.9791514	0.7761399	98.06456
**Gower + Hierarchical clustering**	0.9876818	0.7585090	99.17987
**Breast cancer (imputed dataset)**			
**ClustAll**	0.9610902	0.8380870	97.94083
**Correlation +** **K-means**	0.9968241	0.6030942	99.65134
**Correlation +** **Hierarchical clustering**	0.4000000	0.5632432	46.67692
**Gower +** **K-medoids**	0 9844659	0.7762693	98.21702
**Gower + Hierarchical clustering**	0.9982480	0.7636139	99.41920
**Heart attack (complete dataset)**			
**ClustAll**	0.7983962	0.8041677	85.78361
**Correlation +** **K-means**	0.9451985	0.3085056	99.94616
**Correlation +** **Hierarchical clustering**	0.9504436	0.3008140	99.29759
**Gower +** **K-medoids**	0.8760309	0.6839263	99.18883
**Gower + Hierarchical clustering**	0.8243879	0.2876944	83.26800

ClustAll demonstrated a higher accuracy compared to standard clustering algorithms on case-study datasets, showcasing its robustness and reliability. In both the complete and imputed breast cancer datasets, ClustAll achieved high sensitivity and specificity, having an optimal balance in accurately identifying positive and negative cases. Furthermore, ClustAll exhibited cluster stability, especially with imputed data, indicating consistent and reliable results. Unlike other methods that may overfit or underperform in sensitivity and specificity, ClustAll offers exceptional performance without requiring a predefined number of clusters, making it the most effective and versatile option for clustering tasks.

### 3.4 Computational efficiency

Two primary factors determine the computational efficiency of ClustAll. First, the *number of variables* considered in the *input data* impacts the number of depths of the dendrogram computed during the DCR step. To manage computational load and reduce Central Processing Unit (CPU) time, ClustAll offers a hyperparameter that allows calculation on a subset of depths rather than all, which is particularly useful for initial or exploratory findings. For more comprehensive and consistent results, however, it is advisable to calculate across all possible dendrogram depths.

**Fig 3:**
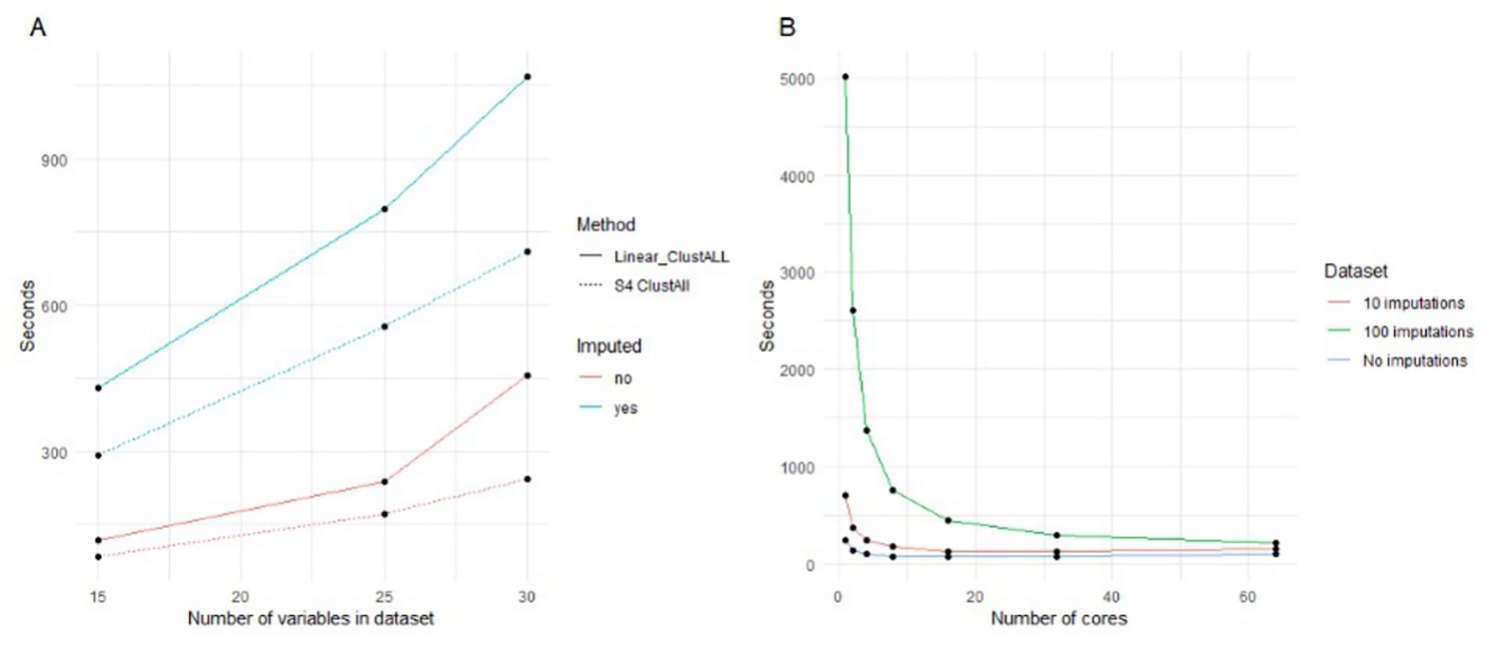
Runtime of ClustAll across different numbers of variables and cores. **(A)** Runtime (in seconds) of two methods ("Linear_ClustALL" and "S4 ClustAll") for different dataset sizes (number of variables) in the breast cancer dataset. This comparison evaluates the sequential (non-parallelized) performance of ClustALL linear and S4 ClustAll using only 1 core. The results are shown for datasets with 10 imputations and without imputations. **(B)** Runtime (in seconds) of S4 ClustAll across different numbers of computational cores for different imputation scenarios (no imputation, 10 imputations, and 100 imputations) on the same dataset. The workstation used for the benchmarking has an AMD EPYC 7742 64-Core Processor with 64 cores and 2,1 TB of RAM.

Second, handling *missing values* in the input data affects the processing time. ClustAll uses multiple imputations to enhance result reliability and prevent dependency on a single imputation. While this approach improves robustness, it also increases computational demand. To address this, parallel computing is incorporated to mitigate the extended processing times associated with multiple imputations. To assess the computational efficiency of parallelized ClustAll, we compared its performance against the linear version of ClustALL with and without imputations. We used the breast cancer dataset [20], varying the dataset size to test scalability. Each method was implemented with optimized parameters and ran multiple times to ensure the reliability of results (**Fig 3A**); we observed a significant decrease in the CPU time using parallelized ClustAll in both approaches.

Additionally, in our benchmarking, using the breast cancer dataset, we demonstrated that utilizing up to 64 cores for 100 imputations increased processing speed by a factor of 22.3 (**Fig 3B**). All approaches in both analyses were run on the same hardware configuration to ensure consistency.

## 4 Availability and future directions

The novel implementation of ClustALL [5], ClustALL, offers a robust and flexible solution for patient stratification, particularly in the context of complex diseases where diverse patient subgroups may exist. While addressing common challenges in clinical data, such as mixed data types, missing values, and collinearity, ClustALL also outperforms traditional clustering algorithms when considering together sensitivity, specificity, and stability. Furthermore, handling missing data through multiple imputations and integrating parallel computing significantly enhances ClustALL’s computational efficiency, making it suitable for large-scale studies. Furthermore, ClustALL’s capability to identify one or more robust stratifications within the same population and to thoroughly evaluate them enables more precise and solid subgroup identification–a critical aspect for advancing precision medicine. Applications of ClustALL to public datasets, such as breast cancer and heart failure data, demonstrated its versatility and potential for broad applicability across various clinical scenarios. The findings show that ClustALL enhances the accuracy of patient stratification and offers a comprehensive framework for exploring and interpreting complex clinical datasets. In summary, ClustALL is a valuable tool for the research community, providing a sophisticated approach to patient stratification that can improve precision treatment and management strategies in clinical practice.

ClustALL is available on Bioconductor: https://bioconductor.org/packages/release/bioc/html/ClustAll.html

Open-source code is freely available at: https://github.com/TranslationalBioinformaticsUnit/ClustAll

## Supporting information

S1 Text This document presents the data analysis and results obtained by applying ClustALL to the two clinical datasets described in the Results Section. Fig A. Heatmap with the Jaccard indexes for population-robust stratifications. Fig B. The Sankey plot shows the distribution and flow of patients between a pair of stratifications. Fig C. The Sankey plot shows the distribution and flow of patients between a stratification and the true labels. Fig D. Heatmap with the Jaccard indexes for population-robust stratifications. Fig E. Heatmap with the Jaccard indexes for population-robust stratifications. Fig F. Heatmap with the Jaccard indexes for population-robust stratifications. Fig G. Heatmap with the Jaccard indexes for population-robust stratifications.
(DOCX)
