# Supplementary material for: ClustAll: An R package for patient stratification in complex diseases
Source: PLoS Comput Biol. 2024 Dec 13;20(12):e1012656. doi: 10.1371/journal.pcbi.1012656 (PMC11676920; doi:10.1371/journal.pcbi.1012656)
Supplement: S1 Text — Fig A. Heatmap with the Jaccard indexes for population-robust stratifications. Fig B. The Sankey plot shows the distribution and flow of patients between a pair of stratifications. Fig C. The Sankey plot shows the distribution and flow of patients between a stratification and the true labels. Fig D. Heatmap with the Jaccard indexes for population-robust stratifications. Fig E. Heatmap with the Jaccard indexes for population-robust stratifications. Fig F. Heatmap with the Jaccard indexes for population-robust stratifications. Fig G. Heatmap with the Jaccard indexes for population-robust stratifications. (DOCX) [file pcbi.1012656.s001.docx]

ClustAll’s User Guide

**Table of Contents**

[1. Introduction ii](#_Toc182644560)

[1.1. Context ii](#_Toc182644561)

[1.2. ClustAll Key Features ii](#_Toc182644562)

[2. Installation ii](#_Toc182644563)

[3. ClustAllObject Class description iii](#_Toc182644564)

[4. ClustAll methods description iv](#_Toc182644565)

[5. Interpreting ClustAll stratification output viii](#_Toc182644566)

[6. Application Example 1 ix](#_Toc182644567)

[7. Application Example 2 xxiv](#_Toc182644568)

[8. References xxviii](#_Toc182644569)

[Benchmarking code xxix](#_Toc182644570)

# 1. Introduction

ClustAll is the *R-Bioconductor* package implementation of the ClustALL algorithm, designed for patient stratification in complex diseases (1).

## Context

In the era of precision medicine, it is necessary to understand heterogeneity among patients with complex diseases to improve personalized prevention and management strategies. Here, we introduce ClustAll, a Bioconductor package designed for unsupervised patient stratification using clinical data. ClustAll is based on the previously validated methodology ClustALL (1), a clustering framework that effectively handles intricacies in clinical data, including mixed data types, missing values, and collinearity. Additionally, ClustAll stands out in its ability to identify multiple patient stratifications within the same population while ensuring their robustness.

## ClustAll Key Features

- **Handles Diverse Data Types**, including missing values, mixed data, and correlated variables.
- **Indentify Robust stratifications**, considering two aspects of robustness **(I)** population-based robustness –an evaluation of stratification stability through bootstrapping – and **(II)** parameter-based robustness – which assesses the stability of the stratification under varied parameter alterations, such as dissimilarity metric or clustering method.
- **Enable the identification of one or more stratifications,** acknowledging that a patient population can be grouped in different ways yet robustly.
- Allows **comparing stratification results with known labels** if they are available.
- **Visualization** functions for interpreting clustering results and comparing different stratifications.

# 2. Installation

ClustAll was developed using S4 object-oriented programming and requires R (>=4.2.0). ClustAll utilizes other R packages currently available from CRAN and Bioconductor, which are included in the DESCRIPTION file.

ClustAll package installation:

**if** (!require("BiocManager", quietly = TRUE)) install.packages("BiocManager")
BiocManager::install("ClustAll")

After installation, load the ClustAll package:

library(ClustAll)

# 3. ClustAllObject Class description

The ClustAllObject class is a core component of the ClustAll package, designed to store and manage data and results throughout the patient stratification process. It is implemented as an S4 class with the following slots:

- *data*: A data frame containing the input data after preprocessing, including one-hot encoding for categorical variables and removing the validation “true labels” column if present.
- *dataOriginal*: The original input data frame is preserved in its unmodified form.
- *dataImputed*: If imputation was applied, this slot contains a mids object from the *mice* package with the imputed datasets. If no imputation was performed, this is *NULL*.
- *dataValidation*: A vector containing the reference labels of the original dataset provided in the data argument. If *NULL*, these labels serve as the "true labels" throughout the analysis. This slot is set to *NULL* by default, meaning no reference labels are provided unless specified. The validation data can be added during object creation via the colValidation parameter in createClustAll, or later using the addValidationData function. These true labels, when available, are used for validation purposes and are not used in the stratification process itself.
- *nImputation*: An integer indicating the number of imputations performed. This is relevant when dealing with missing data.
- *processed*: A boolean flag. *TRUE* denotes that runClustAll has been executed on the object; therefore, the object contains stratification results. *FALSE* otherwise.
- *summary_clusters*: A list containing the resulting stratifications for each combination of clustering methods (distance metric + clustering algorithm) and embedding depth. This is populated after runClustAll has been executed. Otherwise, it is *NULL*.
- *JACCARD_DISTANCE_F*: A matrix of Jaccard distances between the robust stratifications that passed the bootstrapping process. This is used to assess the similarity between different stratification solutions.

This structure allows the ClustAllObject to efficiently store and manage all aspects of the data and results throughout the ClustAll pipeline, from initial data input through preprocessing, imputation (if needed), stratification, and final analysis of results.

# 4. ClustAll methods description

The ClustAll workflow consists of several key functions and methods that enable patient stratification from high-dimensional clinical data. Data may include multiple data types and missing values. The primary functions and methods are outlined next.

**createClustAll**

*createClustAll* function is responsible for creating an S4 object of class *ClustAllObject*. This object is specifically designed to facilitate the application of the ClustAll algorithm, serving as a container for both the process and the primary results of the clustering analysis.

**Usage:**

createClustAll(data, nImputation, dataImputed, colValidation)

**Arguments:**

- *data*: A data frame containing the original dataset. This dataset may include missing values (NAs).
- *nImputation*: A numeric value indicating the number of imputations to be performed if the original dataset contains missing values (NAs). Default is *NULL*, meaning either there are no imputations or mids dataImputed have been provided.
- *dataImputed*: A mids object created using the mice R package, representing the imputed dataset. Both *data* and *dataImputed* must originate from the same source and include identical variables. The default is *NULL*, meaning no imputed data is provided unless specified.
- *colValidation*: A vector containing the reference labels of the original dataset provided in data. If available, these labels will serve as the *true labels* throughout the remainder of the vignette. The default is *NULL*, meaning no reference labels are provided unless specified. This information can be added later using addValidationData.

**Return:**

An unprocessed ClustAllObject without stratification results. Use runClustAll on this object to execute the ClustAll pipeline.

**runClustAll**

The *runClustAll* function executes the ClustALL algorithm. Depending on the characteristics of the data and the clustering results, it produces either none, one, or multiple patient stratifications as output. All generated stratifications are stored in the summary_clusters slot of the ClustAllObject, regardless of their robustness. The JACCARD_DISTANCE_F slot is then filled with a matrix of Jaccard distances between the stratifications that pass the bootstrapping process (population-based robustness). This matrix represents the similarity between robust stratifications and is used for further analysis and visualization.

**Usage:**

runClustAll(Object, threads, simplify)

**Arguments:**

- *Object*: An unprocessed ClustAllObject created by createClustAll. This object contains the input data and any preprocessing, but no stratification results yet. The processed slot of this object should be *FALSE*, indicating that runClustAll has not been previously executed on it.
- *threads*: An integer specifying the number of cores for parallel computing. The default is 1, meaning the process will run on a single core. Adjusting this value can speed up computation by leveraging multiple cores.
- *simplify*: A logical value. If *TRUE*, only every fourth depth of the dendrogram will be considered for the Data Complexity Reduction and Stratification process steps. This reduces execution time and provides preliminary results more quickly. If set to *FALSE*, the function will consider all possible depths of the dendrogram for a more thorough and detailed clustering analysis. The default is *FALSE*.

**Return:**

A processed ClustAllObject containing stratification results from the ClustALL pipeline.

**plotJACCARD**

The *plotJACCARD* function generates a correlation matrix heatmap to visualize the Jaccard Distance between robust population stratifications obtained from the output of the runClustAll function. This visual tool helps in understanding the similarity between different stratifications.

For exploring stratification, it is recommended to begin with a high value for *stratification_similarity* and gradually decrease it as you examine the various clusters generated.

**Usage:**

plotJACCARD(Object, paint, stratification_similarity)

**Arguments:**

- *Object*: A processed ClustAllObject containing stratification results from the ClustALL pipeline. runClustAll needs to be executed to process the ClustAllObject.
- *paint*: A logical value. If set to *TRUE*, groups of similar stratifications (based on the Jaccard Distance) are highlighted with a red square on the heatmap. This helps identify clusters of stratifications with high similarity. The default is *TRUE*.
- *stratification_similarity*: A numeric value that sets the minimum Jaccard Distance required to classify a pair of stratifications as similar. The default value is 0.7, meaning that stratifications with a Jaccard Distance of 0.7 or higher are considered similar.

**Return:**

A plot displaying a correlation matrix heatmap showing the Jaccard Distances between population-based robust stratifications. The heatmap visually distinguishes groups of similar stratifications according to the specified *stratification_similarity* threshold.

**resStratification**

The *resStratification* function retrieves representative stratifications and associated clusters from a processed ClustAllObject. These are based on population and parameter-based robustness analyses using the output generated from the runClustAll function.

**Usage:**

resStratification(Object, population, all, stratification_similarity)

**Arguments**:

- *Object*: A processed ClustAllObject containing stratification results from the ClustALL pipeline. runClustAll needs to be executed to process the ClustAllObject.
- *population*: A numeric value specifying the minimum percentage of the total population that a cluster within a stratification must cover for the stratification to be considered representative. The default is set to 0.05 (5%), meaning a stratification will only be considered if one of its clusters includes at least 5% of the total population.
- *all*: A logical value. If set to *TRUE*, the function will return all stratification representatives that meet the robustness criteria of each group of similar stratifications. If set to *FALSE*, only the centroid stratification (the central that serves as the representative stratification) for each group of similar stratifications will be returned.
- *stratification_similarity*: A numeric value that sets the minimum Jaccard Distance required to classify a pair of stratifications as similar. The default value is 0.7, meaning that stratifications with a Jaccard Distance of 0.7 or higher are considered similar.

**Return:**

A list of robust stratifications and their associated clusters. This list is generated based on the processed *ClustAllObject* after grouping stratifications using the Jaccard Distance and the *stratification_similarity* threshold. For each group of stratifications, all or only centroids will be returned depending on the *all* argument setting.

**plotSANKEY**

The *plotSankey* function generates a Sankey diagram to visually compare the composition of clusters between two stratifications or between a selected stratification and the *true labels* (if available). This visualization provides insight into how the clusters align/differ between stratifications or relative to the reference data.

**Usage:**

plotSankey(Object, clusters, validationData)

**Arguments:**

- *Object*: A processed ClustAllObject containing stratification results from the ClustALL pipeline. runClustAll needs to be executed to process the ClustAllObject.
- *clusters*: character vector specifying the names of the two stratifications to be compared. If validationData is set to *TRUE*, only one stratification name is required in the vector, representing the stratification to be compared with the true labels.
- *validationData*: A logical value. If set to *TRUE*, the function will compare the selected stratification in clusters with the *true labels* (if available). The default is *FALSE*, meaning two stratifications will be compared with each other unless otherwise specified.

**Return:**

A Sankey plot showing the composition and flow of clusters between the selected stratifications. If *true labels* are available and validationData is *TRUE*, the plot will compare the selected stratification against the *true labels*.

**cluster2data**

The *cluster2data* function returns the original dataset, with the selected stratification(s) appended as additional columns. This allows the user to examine the original data alongside the cluster assignments generated by the ClustALL algorithm.

**Usage:**

cluster2data(Object, stratificationName)

**Arguments:**

- *Object*: A processed ClustAllObject containing stratification results from the ClustALL pipeline. runClustAll needs to be executed to process the ClustAllObject.
- *stratificationName*: A character vector specifying one or more stratification names to append to the original dataset as additional columns.

**Return:**

A data frame that includes the original data with additional column(s) containing the selected stratification(s). Each selected stratification will be added as a separate column to the original dataset.

**validateStratification**

The *validateStratification* function validates the results of a selected stratification by comparing it with the *true labels*, if available. *True labels* refer to the validation data from the original dataset. This comparison provides insight into the stratification’s performance using sensitivity and specificity metrics.

Note: To validate the results, ensure that the *colValidation* contains the *true* *labels* vector when applying the *createClustAll* function. Alternatively, *true labels* can be added later using the addValidationData function.

**Usage:**

validateStratification(Object, stratificationName)

**Arguments:**

- *Object*: A processed ClustAllObject containing stratification results from the ClustALL pipeline. runClustAll needs to be executed to process the ClustAllObject.
- *stratificationName*: A character vector containing the name of the stratification to be validated against the true labels.

**Return:**

A numeric vector containing the sensitivity and specificity values of the selected stratification.

# 5. Interpreting ClustAll stratification output

The labels of ClustAll stratification outputs follow a specific format: a letter followed by a number (e.g., "cuts_a_9"). The letter represents the combination of the distance metric and clustering method used to generate that particular stratification (Table 1). The number corresponds to the embedding derived from the depth at which the dendrogram, grouping the variables, was cut. Each stratification represents a solution with both statistical stability and methodological consistency. This labelling convention provides a quick reference to the method and the hierarchical level at which the stratification was produced.

# 6. Application Example 1

6.1. Breast Cancer Wisconsin Dataset Description

ClustAll-package includes two published datasets. One is a published breast cancer dataset of 659 patients (2). This dataset comprises 30 numerical features derived from digitized imaging of a fine needle aspirate (FNA) of a breast mass. Additionally, it includes a categorical variable representing the clinical classification: “malignant” or “benign”.

The breast cancer dataset includes the following features:

1. **radius:** Mean distances from the center to points on the perimeter.
2. **texture:** Standard deviation of gray-scale values.
3. **perimeter:** Perimeter of the breast mass affected by the cancer.
4. **area:** Area of the breast mass affected by the cancer
5. **smoothness:** Local variation in radius lengths.
6. **compactness:** (Perimeter^2 / Area) - 1.0.
7. **concavity:** Severity of concave portions of the contour.
8. **concave points:** Number of concave portions of the contour.
9. **symmetry:** Degree of symmetry in the shape and structure of the breast mass, with higher values indicating higher symmetry and lower values indicating asymmetry.
10. **fractal dimension:** “Coastline approximation” - 1.

The dataset also includes the patient ID and diagnosis (M = malignant, B = benign).

To showcase ClustAll’s effectiveness in handling missing data in a stratification scenario, a modified dataset was generated by randomly replacing values with missing values (NAs).

We denote the original data set as BreastCancerWisconsin (wdbc) and the one with missing values as BreastCancerWisconsinMISSING (wdbcNA).

6.2. Breast Cancer Wisconsin dataset stratification

6.2.1 Get the data

We load the original breast cancer dataset “BreastCancerWisconsin” (wdbc= winconsin database cancer). The dataset comprises 30 numerical features. As the dataset does not contain missing values, we will apply the ClustAll workflow accordingly.

*# load example data*data("BreastCancerWisconsin", package = "ClustAll") *# remove patients IDs (non-informative).*data_use <- subset(wdbc, select = -ID) *# explore the features of example data*str(data_use)

## 'data.frame': 569 obs. of 31 variables:
## $ Diagnosis : chr "M" "M" "M" "M" ...
## $ radius1 : num 18 20.6 19.7 11.4 20.3 ...
## $ texture1 : num 10.4 17.8 21.2 20.4 14.3 ...
## $ perimeter1 : num 122.8 132.9 130 77.6 135.1 ...
## $ area1 : num 1001 1326 1203 386 1297 ...
## $ smoothness1 : num 0.1184 0.0847 0.1096 0.1425 0.1003 ...
## $ compactness1 : num 0.2776 0.0786 0.1599 0.2839 0.1328 ...
## $ concavity1 : num 0.3001 0.0869 0.1974 0.2414 0.198 ...
## $ concave_points1 : num 0.1471 0.0702 0.1279 0.1052 0.1043 ...
## $ symmetry1 : num 0.242 0.181 0.207 0.26 0.181 ...
## $ fractal_dimension1: num 0.0787 0.0567 0.06 0.0974 0.0588 ...
## $ radius2 : num 1.095 0.543 0.746 0.496 0.757 ...
## $ texture2 : num 0.905 0.734 0.787 1.156 0.781 ...
## $ perimeter2 : num 8.59 3.4 4.58 3.44 5.44 ...
## $ area2 : num 153.4 74.1 94 27.2 94.4 ...
## $ smoothness2 : num 0.0064 0.00522 0.00615 0.00911 0.01149 ...
## $ compactness2 : num 0.049 0.0131 0.0401 0.0746 0.0246 ...
## $ concavity2 : num 0.0537 0.0186 0.0383 0.0566 0.0569 ...
## $ concave_points2 : num 0.0159 0.0134 0.0206 0.0187 0.0188 ...
## $ symmetry2 : num 0.03 0.0139 0.0225 0.0596 0.0176 ...
## $ fractal_dimension2: num 0.00619 0.00353 0.00457 0.00921 0.00511 ...
## $ radius3 : num 25.4 25 23.6 14.9 22.5 ...
## $ texture3 : num 17.3 23.4 25.5 26.5 16.7 ...
## $ perimeter3 : num 184.6 158.8 152.5 98.9 152.2 ...
## $ area3 : num 2019 1956 1709 568 1575 ...
## $ smoothness3 : num 0.162 0.124 0.144 0.21 0.137 ...
## $ compactness3 : num 0.666 0.187 0.424 0.866 0.205 ...
## $ concavity3 : num 0.712 0.242 0.45 0.687 0.4 ...
## $ concave_points3 : num 0.265 0.186 0.243 0.258 0.163 ...
## $ symmetry3 : num 0.46 0.275 0.361 0.664 0.236 ...
## $ fractal_dimension3: num 0.1189 0.089 0.0876 0.173 0.0768 ...

6.2.2 Create the input S4 object ClustAllObject

First, the ClustAllObject object is created using the createClustAll function. In this step, we indicate that “Diagnosis” is the feature that contains the *true labels* in the argument *colValidation*. Note that the *true labels* are stored separately in *dataValidation* slot and are not used to compute stratifications; they are only used later for validation purposes..

obj_noNA <- createClustAll(data = data_use, nImputation = NULL,
 dataImputed = NULL, colValidation = "Diagnosis")

## The dataset contains character values.

## They are converted to categorical (more than one class) or to binary (one class).

## Before continuing, check that the transformation has been processed correctly.

##
## ClustALL object created successfully. You can use runClustAll.

6.2.3 Execute the ClustALL algorithm

Next, we apply the ClustALL algorithm executing the runClustAll function. The output is stored in a ClustAllObject, which contains the clustering results.

obj_noNA1 <- runClustAll(Object = obj_noNA, threads = 2, simplify = FALSE)

## ______ __ __ ___ __ __

## / ____// /__ __ _____ / /_ / | / / / /

## / / / // / / // ___// __// /| | / / / /

## / /___ / // /_/ /(__ )/ /_ / ___ | / /___ / /___

## /_____//_/ |__,_//____/ |__//_/ |_|/_____//_____/

## Running Data Complexity Reduction and Stratification Process.

## This step may take some time...

## Calculating correlation distance matrix of the stratifications...

## Filtering non-robust stratifications...

## ClustAll pipeline finished successfully!

obj_noNA1

*## ClustAllObject*

*## Data: Number of variables: 30. Number of patients: 569*

*## Imputated: NO.*

*## Number of imputations: 0*

*## Processed: TRUE*

*## Number of stratifications: 72*

After executing runClustAll, we obtain the processed ClustAllObject. Printing it to the screen provides a summary of its contents. The initial dataset includes 30 variables and 589 patients, with no imputations applied. The object has been processed and contains 72 stratifications.

6.2.4 Jaccard Distance between population-robust stratifications

To depict the similarity between population-robust stratifications (those with >85% bootstrapping stability), a heatmap representation of the Jaccard index between stratifications is provided. This visualization helps in comparing the overlap of clustering solutions. Discontinuous red rectangles highlight alternative stratification solutions based on a predefined similarity threshold (ranging from 0 to 1; default 0.7) specified in the *stratification_similarity* argument. The row annotations in the heatmap detail the parameters used to derive each stratification, including the distance metric, clustering method, and the embedding number (depth of dendrogram cut).

For exploratory analysis using the plotJACCARD function, we recommend starting with a high *stratification_similarity* threshold and gradually decreasing it if needed. In our case studies, we initially used a threshold of 0.9 to identify highly similar stratification groups. However, if no informative results are obtained at this strict threshold, users can iteratively lower the similarity threshold to be less restrictive until meaningful stratification groups emerge. This approach allows users to prioritize the most robust and consistent stratifications before considering more diverse or less stable solutions. The default value of the *stratification_similarity* parameter is 0.7, which is a good starting point for most analyses as it is not very restrictive. The optimal value can vary greatly depending on the dataset, so users should adjust this value according to the specific characteristics of their data and the desired granularity of the stratification groups.

In this specific case, a Jaccard similarity of 0.9 reveals three different groups of alternatives for robustly stratifying the population. These stratifications are depicted by the discontinuous red squares (**Fig A**). **(i)** The first stratification option is derived from 8 alternatives, which includes correlation distance with K-means and Gower with K-medoids, along with different embedding depths; **(ii)** The second option is derived from 32 alternatives based on correlation and Gower distances with K-means and K-medoids, along with different embedding depths; **(iii)** The third option comprises 5 instances, derived from correlation distance with K-medoids and different embeddings depths.

plotJACCARD(Object = obj_noNA1, stratification_similarity = 0.9)

**Fig A. Heatmap with the Jaccard indexes for population-robust stratifications.** Heatmap representing the similarity between stratifications using the Jaccard Index.  It groups similar stratifications, allowing for the identification of patterns that exhibit similar behavior. The X-axis and Y-axis represent the different stratifications. The color gradient, ranging from blue to white, indicates the Jaccard Index values. A darker blue represents a higher Jaccard Index, indicating greater similarity between sets, while a lighter blue (approaching white) represents a lower Jaccard Index, indicating less similarity. Red dashed lines highlight groups of stratifications that show a high degree of similarity based on the Jaccard Index threshold (in this case Jaccard index is 0.9). The label “Distance” refers to the type of similarity measure used, such as Correlation or Gower distance. The label “Clustering” indicates the clustering method applied, such as Hierarchical clustering, k-means, or k-medoids. “Depth” refers to the level of embedding, showing in a color range the depths of the dendrogram. *H-Clustering*: hierarchical clustering.

6.2.5 Retrieve stratification representatives

The centroids (representative stratifications) from each group of alternative stratification solutions, highlighted in red squares in the previous step, are displayed. Each representative stratification provides a summary of the clustering results, showing both the number of clusters and the patients belonging to each cluster. These centroids offer a simplified view of the broader clustering solutions, allowing for a more straightforward interpretation of the overall population structure derived from the alternative stratification methods.

resStratification(Object = obj_noNA1, population = 0.05, stratification_similarity = 0.9, all = FALSE)

## $cuts_a_28
## $cuts_a_28[[1]]
## 1 2
## 183 386
##
## $cuts_c_9
## $cuts_c_9[[1]]
## 1 2
## 197 372
##
## $cuts_c_4
## $cuts_c_4[[1]]
## 1 2
## 199 370

In this case, the alternative stratifications have been computed based on stratification similarity of 0.9, and considering that clusters should comprise a minimum of 5% of the total population:

- cuts_a_28: This stratification was generated using embedding derived from dendrogram depth 28 with the correlation distance metric and the k-means clustering algorithm. It consists of two clusters, with 183 and 386 patients, respectively.
- cuts_c_9: This stratification was generated using embedding derived from dendrogram depth 9 with the Gower distance metric and the k-medoids clustering algorithm. It consists of two clusters, with 197 and 372 patients, respectively.
- cuts_c_4: This stratification was generated using embedding derived from dendrogram depth 4 with the Gower distance metric and the k-medoids clustering algorithm. It consists of two clusters, with 199 and 370 patients, respectively.

6.2.6 Sankey diagrams comparing pairs of stratifications

Sankey plots allow illustrate the differences between clusters of alternative stratifications or compare a stratification with the *true labels* (in this case, the clinical diagnosis is not used during the stratification process).

In this analysis:

- The first Sankey plot **(Fig B)** shows patient transitions between two sets of stratification representatives (cuts_a_28 and cuts_c_9). This plot highlights the flow and distribution of patients across the clusters, offering insight into how different stratification methods assign individuals to clusters.
- The second Sankey plot **(Fig C)** shows the patient transitions between a stratification representative (cuts_a_28) and the true labels (i.e., clinical diagnosis). This plot reveals the alignment (or misalignment) between the stratification derived from the ClustALL algorithm and the true diagnostic labels, showcasing how well the clustering method corresponds with the actual clinical classifications.

plotSANKEY(Object = obj_noNA1, clusters = c("cuts_a_28", "cuts_c_9"),
 validationData = FALSE)

**

**Fig B. The Sankey plot shows the distribution and flow of patients between a pair of stratifications.** The blue bars (left) represent the two groups corresponding to the “cuts_a_28“ stratification, and the orange bars (right) represent the two groups corresponding to the “cuts_c_9“ stratification.

plotSANKEY(Object = obj_noNA1, clusters = c("cuts_a_28"), validationData = TRUE)

**

**Fig C. The sankey plot shows the distribution and flow of patients between a stratification and the true labels.** The blue bars (left) represent the two groups corresponding to the “cuts_a_28“ stratification, and the orange bars (right) represent the two groups corresponding to the true labels (reference from the original data) that allow the validation of the results.

6.2.7 Retrieve the original dataset with the selected ClustAll stratifications

The three stratification representatives are added to the initial dataset.

df <- cluster2data(Object = obj_noNA1, stratificationName = c("cuts_a_28",
 "cuts_c_9", "cuts_c_4"))

head(df, 3)

## radius1 texture1 perimeter1 area1 smoothness1 compactness1 concavity1
## 1 17.99 10.38 122.8 1001 0.11840 0.27760 0.3001
## 2 20.57 17.77 132.9 1326 0.08474 0.07864 0.0869
## 3 19.69 21.25 130.0 1203 0.10960 0.15990 0.1974
## concave_points1 symmetry1 fractal_dimension1 radius2 texture2 perimeter2
## 1 0.14710 0.2419 0.07871 1.0950 0.9053 8.589
## 2 0.07017 0.1812 0.05667 0.5435 0.7339 3.398
## 3 0.12790 0.2069 0.05999 0.7456 0.7869 4.585
## area2 smoothness2 compactness2 concavity2 concave_points2 symmetry2
## 1 153.40 0.006399 0.04904 0.05373 0.01587 0.03003
## 2 74.08 0.005225 0.01308 0.01860 0.01340 0.01389
## 3 94.03 0.006150 0.04006 0.03832 0.02058 0.02250
## fractal_dimension2 radius3 texture3 perimeter3 area3 smoothness3 compactness3
## 1 0.006193 25.38 17.33 184.6 2019 0.1622 0.6656
## 2 0.003532 24.99 23.41 158.8 1956 0.1238 0.1866
## 3 0.004571 23.57 25.53 152.5 1709 0.1444 0.4245
## concavity3 concave_points3 symmetry3 fractal_dimension3 cuts_a_28 cuts_c_9
## 1 0.7119 0.2654 0.4601 0.11890 1 1
## 2 0.2416 0.1860 0.2750 0.08902 1 1
## 3 0.4504 0.2430 0.3613 0.08758 1 1
## cuts_c_4
## 1 1
## 2 1
## 3 1

6.2.8 Assess the sensitivity and specificity of the selected ClustAll stratifications

The sensitivity and specificity parameters are calculated to assess the performance of the identified ClustAll stratifications against the true labels (if available). Higher values of these metrics indicate greater precision in the stratification process, reflecting how well the clustering aligns with the actual classifications.

In this case, all three stratification representatives show sensitivity and specificity values exceeding 80% and 90%, respectively, despite being derived from different clustering methods. These results highlight the robustness of the stratification process, demonstrating that consistent and accurate groupings can be achieved across various clustering techniques.

*# STRATIFICATION 1*validateStratification(obj_noNA1, "cuts_a_28")

## sensitivity specificity

## 0.8207547 0.9747899

*# STRATIFICATION 2*validateStratification(obj_noNA1, "cuts_c_9")

## sensitivity specificity
## 0.8584906 0.9579832

*# STRATIFICATION 3*validateStratification(obj_noNA1, "cuts_c_4")

## sensitivity specificity
## 0.8820755 0.9663866

6.3 Breast Cancer Wisconsin with missing data imputed within ClustAll framework

6.3.1 Get the data

data("BreastCancerWisconsinMISSING", package = "ClustAll")
data_use_NA <- wdbcNA
summary(data_use_NA) *# dataset present NAs*

## Diagnosis radius1 texture1 perimeter1 area1
## B:357 Min. : 6.981 Min. : 9.71 Min. : 43.79 Min. : 143.5
## M:212 1st Qu.:11.707 1st Qu.:16.17 1st Qu.: 75.17 1st Qu.: 420.3
## Median :13.355 Median :18.84 Median : 86.24 Median : 551.1
## Mean :14.077 Mean :19.29 Mean : 91.97 Mean : 654.9
## 3rd Qu.:15.750 3rd Qu.:21.80 3rd Qu.:104.10 3rd Qu.: 782.7
## Max. :27.420 Max. :39.28 Max. :188.50 Max. :2501.0
## NA's :17
## smoothness1 compactness1 concavity1 concave_points1
## Min. :0.05263 Min. :0.01938 Min. :0.00000 Min. :0.00000
## 1st Qu.:0.08637 1st Qu.:0.06492 1st Qu.:0.02903 1st Qu.:0.02030
## Median :0.09587 Median :0.09263 Median :0.05988 Median :0.03345
## Mean :0.09636 Mean :0.10434 Mean :0.08828 Mean :0.04861
## 3rd Qu.:0.10530 3rd Qu.:0.13040 3rd Qu.:0.13000 3rd Qu.:0.07346
## Max. :0.16340 Max. :0.34540 Max. :0.42680 Max. :0.20120
## NA's :34 NA's :13
## symmetry1 fractal_dimension1 radius2 texture2
## Min. :0.1060 Min. :0.04996 Min. :0.1115 Min. :0.3602
## 1st Qu.:0.1619 1st Qu.:0.05770 1st Qu.:0.2324 1st Qu.:0.8355
## Median :0.1792 Median :0.06154 Median :0.3242 Median :1.0950
## Mean :0.1812 Mean :0.06280 Mean :0.4052 Mean :1.2196
## 3rd Qu.:0.1957 3rd Qu.:0.06612 3rd Qu.:0.4789 3rd Qu.:1.4740
## Max. :0.3040 Max. :0.09744 Max. :2.8730 Max. :4.8850
## NA's :40
## perimeter2 area2 smoothness2 compactness2
## Min. : 0.757 Min. : 6.802 Min. :0.001713 Min. :0.002252
## 1st Qu.: 1.606 1st Qu.: 17.850 1st Qu.:0.005169 1st Qu.:0.012745
## Median : 2.287 Median : 24.530 Median :0.006380 Median :0.020450
## Mean : 2.866 Mean : 40.337 Mean :0.007041 Mean :0.025390
## 3rd Qu.: 3.357 3rd Qu.: 45.190 3rd Qu.:0.008146 3rd Qu.:0.032130
## Max. :21.980 Max. :542.200 Max. :0.031130 Max. :0.135400
## NA's :30
## concavity2 concave_points2 symmetry2 fractal_dimension2
## Min. :0.00000 Min. :0.000000 Min. :0.00788 Min. :0.0008948
## 1st Qu.:0.01509 1st Qu.:0.007711 1st Qu.:0.01519 1st Qu.:0.0022480
## Median :0.02589 Median :0.011090 Median :0.01879 Median :0.0031870
## Mean :0.03189 Mean :0.011873 Mean :0.02066 Mean :0.0037949
## 3rd Qu.:0.04205 3rd Qu.:0.014940 3rd Qu.:0.02354 3rd Qu.:0.0045580
## Max. :0.39600 Max. :0.052790 Max. :0.07895 Max. :0.0298400
## NA's :20 NA's :34
## radius3 texture3 perimeter3 area3
## Min. : 8.678 Min. :12.02 Min. : 50.41 Min. : 185.2
## 1st Qu.:13.010 1st Qu.:21.40 1st Qu.: 84.11 1st Qu.: 515.3
## Median :14.960 Median :25.48 Median : 97.66 Median : 686.5
## Mean :16.202 Mean :25.86 Mean :107.26 Mean : 880.6
## 3rd Qu.:18.550 3rd Qu.:29.72 3rd Qu.:125.40 3rd Qu.:1084.0
## Max. :33.130 Max. :49.54 Max. :251.20 Max. :4254.0
## NA's :48 NA's :56
## smoothness3 compactness3 concavity3 concave_points3
## Min. :0.07117 Min. :0.02729 Min. :0.0000 Min. :0.00000
## 1st Qu.:0.11660 1st Qu.:0.14720 1st Qu.:0.1145 1st Qu.:0.06493
## Median :0.13130 Median :0.21190 Median :0.2267 Median :0.09993
## Mean :0.13229 Mean :0.25427 Mean :0.2722 Mean :0.11461
## 3rd Qu.:0.14600 3rd Qu.:0.33910 3rd Qu.:0.3829 3rd Qu.:0.16140
## Max. :0.22260 Max. :1.05800 Max. :1.2520 Max. :0.29100
## NA's :22
## symmetry3 fractal_dimension3
## Min. :0.1565 Min. :0.05504
## 1st Qu.:0.2504 1st Qu.:0.07182
## Median :0.2822 Median :0.08009
## Mean :0.2901 Mean :0.08400
## 3rd Qu.:0.3179 3rd Qu.:0.09215
## Max. :0.6638 Max. :0.20750
## NA's :52

6.3.2 Create the input S4 object ClustAllObject

First, the ClustAllObject object is created. Since the initial data contains missing values ClustAll offers two possible scenarios:

**Automatic imputation**: If the input data contains missing values, ClustAll can automatically perform imputation using the specified number of imputations (applying *mice* package).

**Manual imputation**: In some cases, users may choose to impute missing values manually before creating the ClustAllObject. This approach is particularly useful when users have prior knowledge about the data and the relationships between variables. Manual imputation allows for more tailored and context-specific handling of missing values.

In this specific case, assuming that we do not have prior knowledge of the dataset, we performed an automatic imputation. To limit computational time, the number of imputations was set to 2. However, we recommend a large number of imputations, typically n>=100. In addition, we indicate that “Diagnosis” is the feature that contains the *true labels*, which will be used for validation purposes later in the analysis*.*

obj_NA <- createClustAll(data_use_NA, nImputation = 2, colValidation ="Diagnosis")

## Before continuing, check that the transformation has been processed correctly.

## Running default multiple imputation method.

## For more information check mice package.
## ClustALL object created successfully. You can use runClustAll.

The rest of the pipeline follows as in *section 6.2.3.*

6.3.3 Execute the ClustALL algorithm

The ClustALL algorithm is computed using the runClustAll, and the output is stored in the ClustAllObject.

obj_NA1 <- runClustAll(obj_NA, threads = 2)

## ______ __ __ ___ __ __

## / ____// /__ __ _____ / /_ / | / / / /

## / / / // / / // ___// __// /| | / / / /

## / /___ / // /_/ /(__ )/ /_ / ___ | / /___ / /___

## /_____//_/ |__,_//____/ |__//_/ |_|/_____//_____/

## Running Data Complexity Reduction and Stratification Process.

## This step may take some time...

## Calculating correlation distance matrix of the stratifications...

## Filtering non-robust stratifications...

## ClustAll pipeline finished successfully!

obj_NA1

## ClustAllObject

## Data: Number of variables: 30. Number of patients: 569

## Imputation: YES.

## Number of imputations: 2

## Processed: TRUE

## Number of stratifications: 17

After running runClustAll, we obtain the processed ClustAllObject. Printing it to the screen provides a summary of its contents. The initial dataset includes 30 variables and 589 patients, with 2 imputations applied. The object has been processed and contains 17 stratifications.

6.3.4 Jaccard Distance between population-robust stratifications

In this case, we used a high *stratification_similarity* threshold of 0.9 to prioritize identifying the most robust and consistent stratification groups. By starting with a strict threshold, we ensure that the identified groups are highly similar and likely to represent stable solutions. As shown in **Fig D**, all the robust stratifications are very similar and, therefore, belong to the same group.

plotJACCARD(Object = obj_NA1, stratification_similarity = 0.9)

**Fig D. Heatmap with the Jaccard indexes for population-robust stratifications.** Heatmap representing the similarity between stratifications using the Jaccard index of 0.9.

6.3.5 Retrieve stratification representatives

We then retrieve the stratification representative or centroid, which corresponds to cuts_a_2.

resStratification(Object = obj_NA1, population = 0.05, stratification_similarity = 0.9, all = FALSE)

## $cuts_a_2
## $cuts_a_2[[1]]
##
## 1 2
## 194 375

- cuts_a_2: This stratification was generated using embedding derived from dendrogram depth 2 with the correlation distance metric and the k-means clustering algorithm. It consists of two clusters, with 194 and 375 patients, respectively.

6.3.6 Assess the sensitivity and specificity of the selected ClustAll stratifications

The assessment of the selected ClustAll stratification against the *true labels* reveals a sensitivity and a specificity higher than 80% and 95%, respectively.

validateStratification(obj_NA1, "cuts_a_2")

## sensitivity specificity
## 0.8443396 0.9579832

6.4 Breast Cancer Wisconsin with missing data imputed externally

6.4.1 Get the data and compute imputation

The initial data contains missing values. The imputation is performed independently and stored using the *mice* R package.

library(mice)
data("BreastCancerWisconsinMISSING", package = "ClustAll") *# load example data*data_use_NA <- wdbcNA
str(data_use_NA)

imp_data_use <- mice(data_use_NA[-1], m = 2, maxit = 5, seed = 1234,print = FALSE)

6.4.2 Create the input S4 object ClustAllObject

The ClustAllObject object is then created and stored. The initial data contained missing values but was imputed externally (previous step). To generate the ClustAllObject, we specify the original dataset with missing values (*data*) and the imputed dataset (*dataImputed*). In addition, we indicate that “Diagnosis” is the feature that contains the *true labels* in *colValidation*.

*# dataImputed contains the mids object with the imputed*obj_imp1 <- createClustAll(data = data_use_NA, dataImputed = imp_data_use,
 colValidation = "Diagnosis")

## Before continuing, check that the transformation has been processed correctly.

##
## ClustALL object created successfully. You can use runClustAll.

The rest of the pipeline follows as in section 6.2.3

6.4.3 Execute the ClustALL algorithm

The ClustALL algorithm is computed using the runClustAll, and the output is stored in the ClustAllObject.

obj_imp1 <- runClustAll(obj_imp1, threads = 2)

## ______ __ __ ___ __ __

## / ____// /__ __ _____ / /_ / | / / / /

## / / / // / / // ___// __// /| | / / / /

## / /___ / // /_/ /(__ )/ /_ / ___ | / /___ / /___

## /_____//_/ |__,_//____/ |__//_/ |_|/_____//_____/

## Running Data Complexity Reduction and Stratification Process.

## This step may take some time...

## Calculating correlation distance matrix of the stratifications...

## Filtering non-robust stratifications...

## ClustAll pipeline finished successfully!

obj_imp1

## ClustAllObject

## Data: Number of variables: 30. Number of patients: 569

## Imputation: YES.

## Number of imputations: 2

## Processed: TRUE

## Number of stratifications: 17

After running runClustAll, we obtain the processed ClustAllObject. Printing it to the screen provides a summary of its contents. The initial dataset includes 30 variables and 589 patients, with 2 imputations applied in the externally imputed data. The object has been processed and contains 17 stratifications.

6.4.4 Jaccard Distance between population-robust stratifications

In this specific case, a high *stratification_similarity* value such as 0.9 reveals a unique alternative for stratifying the population, which is comprised of 16 equivalent stratifications (**Fig E**).

plotJACCARD(Object = obj_imp1, stratification_similarity = 0.9)

**Fig E. Heatmap with the Jaccard indexes for population-robust stratifications.** Heatmap representing the similarity between stratifications using the Jaccard index of 0.9.

6.4.5 Retrieve stratification representatives

We then retrieve the stratification representative or centroid, which corresponds to cuts_a_2.

resStratification(Object = obj_imp1, population = 0.05, stratification_similarity = 0.9, all = FALSE)

## $cuts_a_2
## $cuts_a_2[[1]]
##
## 1 2
## 194 375

- cuts_a_2: This stratification was generated using embedding derived from dendrogram depth 2 with the correlation distance metric and the k-means clustering algorithm. It consists of two clusters, with 194 and 375 patients, respectively.

6.4.6 Assess the sensitivity and specificity of the selected ClustAll stratifications

The assessment of the selected ClustAll stratification against the *true labels* reveals a sensitivity and a specificity higher than 80% and 95%, respectively.

validateStratification(obj_imp1, "cuts_a_2")

## sensitivity specificity
## 0.8443396 0.9579832

# 7. Application Example 2

7.1 Heart Disease Dataset Description

A second public dataset was available in the ClustAll package, a heart disease dataset. The dataset was originally designed to develop predictors of heart failure based on clinical test data (3). This dataset includes 918 patient records and 11 clinical features, comprising 6 categorical and 5 numerical variables. It does not contain missing values.

A depiction of the features is described in the following lines:

1. **Age.**
2. **Sex.**
3. **ChestPainType:** Types of chest pain include “Typical Angina (TA)”, “Atypical Angina (ATA)”, “Non-Anginal Pain (NAP)”, and “Asymptomatic (ASY)”.
4. **RestingBP:** Resting blood pressure (mmHg).
5. **Cholesterol:** Serum cholesterol (mm/dl).
6. **FastingBS:** If fasting blood sugar is greater than 120, it is 1; else, it is 0.
7. **RestingECG:** Results of a resting electrocardiograph (ECG) include “Normal (Normal)”, ”Irregular ST-T wave (ST)“, and possible “left ventricular hypertrophy (LVH)”.
8. **MaxHR:** Highest heart rate attained.
9. **ExerciseAngina:** Angina induced by exercise.
10. **Oldpeak:** ST depression induced by exercise relative to rest.
11. **ST_Slope:** The slope of the peak exercise (ST segment).

The dataset also includes the clinical outcome heart failure, represented by the variable **HeartDisease** (0 = No, 1 = Yes), which serves as *true labels.* The variable **HeartDisease** is not included in the input; it is only used to compare against identified stratifications.

7.2 Get the data from the example, exploration, and transformation application

We load the data and check variable types. We convert integer (int) variables to numeric.

data("heart", package = "ClustAll")
str(heart_data)

## 'data.frame': 918 obs. of 12 variables:
## $ Age : int 40 49 37 48 54 39 45 54 37 48 ...
## $ Sex : chr "M" "F" "M" "F" ...
## $ ChestPainType : chr "ATA" "NAP" "ATA" "ASY" ...
## $ RestingBP : int 140 160 130 138 150 120 130 110 140 120 ...
## $ Cholesterol : int 289 180 283 214 195 339 237 208 207 284 ...
## $ FastingBS : int 0 0 0 0 0 0 0 0 0 0 ...
## $ RestingECG : chr "Normal" "Normal" "ST" "Normal" ...
## $ MaxHR : int 172 156 98 108 122 170 170 142 130 120 ...
## $ ExerciseAngina: chr "N" "N" "N" "Y" ...
## $ Oldpeak : num 0 1 0 1.5 0 0 0 0 1.5 0 ...
## $ ST_Slope : chr "Up" "Flat" "Up" "Flat" ...
## $ HeartDisease : int 0 1 0 1 0 0 0 0 1 0 ...

*library(dplyr)*

heart_data <- heart_data %>%
 mutate_at(c("Age", "RestingBP", "Cholesterol", "FastingBS",
 "MaxHR"), as.numeric)
str(heart_data)

## 'data.frame': 918 obs. of 12 variables:
## $ Age : num 40 49 37 48 54 39 45 54 37 48 ...
## $ Sex : chr "M" "F" "M" "F" ...
## $ ChestPainType : chr "ATA" "NAP" "ATA" "ASY" ...
## $ RestingBP : num 140 160 130 138 150 120 130 110 140 120 ...
## $ Cholesterol : num 289 180 283 214 195 339 237 208 207 284 ...
## $ FastingBS : num 0 0 0 0 0 0 0 0 0 0 ...
## $ RestingECG : chr "Normal" "Normal" "ST" "Normal" ...
## $ MaxHR : num 172 156 98 108 122 170 170 142 130 120 ...
## $ ExerciseAngina: chr "N" "N" "N" "Y" ...
## $ Oldpeak : num 0 1 0 1.5 0 0 0 0 1.5 0 ...
## $ ST_Slope : chr "Up" "Flat" "Up" "Flat" ...
## $ HeartDisease : int 0 1 0 1 0 0 0 0 1 0 ...

7.3 Create the input S4 object ClustAllObject

The ClustAllObject object is then created and stored.

obj_heartnoNA <- createClustAll(data = heart_data, colValidation = "HeartDisease")

## The dataset contains character values.

## They are converted to categorical (more than one class) or to binary (one class).

## Categorical variables detected! Applying One Hot encoding...

## Before continuing, check that the transformation has been processed correctly.

##
## ClustALL object created successfully. You can use runClustAll.

The rest of the pipeline follows as in section 6.2.3

7.4 Execute the ClustALL algorithm

The ClustALL algorithm is applied, and the results are stored in a ClustAllObject that contains the clustering results.

obj_heartnoNA1 <- runClustAll(Object = obj_heartnoNA, threads = 2,simplify =FALSE)

## ______ __ __ ___ __ __

## / ____// /__ __ _____ / /_ / | / / / /

## / / / // / / // ___// __// /| | / / / /

## / /___ / // /_/ /(__ )/ /_ / ___ | / /___ / /___

## /_____//_/ |__,_//____/ |__//_/ |_|/_____//_____/

## Running Data Complexity Reduction and Stratification Process.

## This step may take some time...

## Calculating correlation distance matrix of the stratifications...

## Filtering non-robust stratifications...
## ClustAll pipeline finished successfully!

obj_heartnoNA1

## ClustAllObject

## Data: Number of variables: 11. Number of patients: 918

## Imputation: YES.

## Number of imputations: 2

## Processed: TRUE

## Number of stratifications: 18

After running runClustAll, we obtain the processed ClustAllObject. Printing it to the screen provides a summary of its contents. The initial dataset includes 11 variables and 918 patients, with 2 imputations applied. The object has been processed and contains 18 stratifications.

7.5. Jaccard Distance between population-robust stratifications

We represent the Jaccard Distance between population-robust stratifications in a heatmap. In this case, we prioritized consistency using a high *stratification_similarity* of 0.9. This approach ensures that the identified stratification groups are highly robust and stable. While lowering the similarity threshold would yield more stratification groups, it would also introduce less consistent solutions. Our choice of a 0.9 threshold emphasizes the importance of identifying the most reliable and reproducible stratifications for this analysis, resulting in a single alternative comprised of 5 consistent stratifications (**Fig F**). For example, by setting to 0.7 the stratification_similarity, we would obtain an extra cluster less consistent composed of 3 alternatives, and the previous cluster would be composed of additional (less similar) 3 stratifications (**Fig G**).

plotJACCARD(Object = obj_heartnoNA1, stratification_similarity = 0.9)

**Fig F. Heatmap with the Jaccard indexes for population-robust stratifications.** Heatmap representing the similarity between stratifications using a Jaccard index of 0.9.

plotJACCARD(Object = obj_heartnoNA1, stratification_similarity = 0.7)


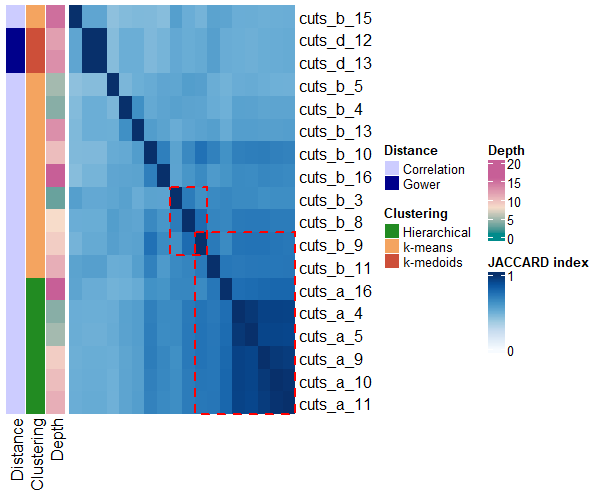


**Fig G. Heatmap with the Jaccard indexes for population-robust stratifications.** Heatmap representing the similarity between stratifications using a Jaccard index of 0.7.

7.6. Retrieve stratification representative(s)

We then retrieve the stratification representative or centroid, which corresponds to cuts_a_9.

resStratification(Object = obj_heartnoNA1, population = 0.05,
 stratification_similarity = 0.9, all = FALSE)

## $cuts_a_9
## $cuts_a_9[[1]]
## 1 2
## 402 516

- cuts_a_9: This stratification was generated using embedding derived from dendrogram depth 9 with the correlation distance metric and the k-means clustering algorithm. It consists of two clusters, with 402 and 516 patients, respectively.

7.7. Assess the sensitivity and specificity of the selected ClustAll stratifications

The assessment of the selected ClustAll stratification against *true labels* reveals a sensitivity and a specificity of 87% and 82%, respectively..

validateStratification(obj_heartnoNA1, "cuts_a_9")

## sensitivity specificity
## 0.8740157 0.8243902

sessionInfo()

## R version 4.3.0 (2023-04-21)
## Platform: aarch64-apple-darwin20 (64-bit)
## Running under: macOS Monterey 12.0
##
## Matrix products: default
## BLAS: /Library/Frameworks/R.framework/Versions/4.3-arm64/Resources/lib/libRblas.0.dylib
## LAPACK: /Library/Frameworks/R.framework/Versions/4.3-arm64/Resources/lib/libRlapack.dylib; LAPACK version 3.11.0
##
## locale:
## [1] en_US.UTF-8/en_US.UTF-8/en_US.UTF-8/C/en_US.UTF-8/en_US.UTF-8
##
## time zone: Europe/Madrid
## tzcode source: internal
##
## attached base packages:
## [1] stats graphics grDevices utils datasets methods base
##
## other attached packages:
## [1] ClustAll_1.0.0 bookdown_0.38 mice_3.16.0 dplyr_1.1.4
## [5] knitr_1.45

# 8. References

1. Palomino-Echeverria S, Huergo E, Ortega-Legarreta A, Uson Raposo EM, Aguilar F, Peña-Ramirez C de la, et al. A robust clustering strategy for stratification unveils unique patient subgroups in acutely decompensated cirrhosis. J Transl Med. 2024 Jun 27;22(1):599.

2. Kaggle. https://www.kaggle.com/datasets/uciml/breastcancer-wisconsin-data. Breast Cancer Wisconsin (Diagnostic) Data Set. .

3. Kaggle. https://www.kaggle.com/datasets/fedesoriano/heart-failure-prediction. Heart Failure Prediction Dataset.

# Benchmarking code

In this section, we provide the code needed to reproduce the ClustAll benchmarking results presented in the main text.

**ClustAll performance against standard clustering algorithms**

##### OTHER METHODS (to compare against CLustAll) #####

# Define clustering functions

# Correlation + K-Means

cluster_function1 <- function(data, k) {

cor_kmeans <- clValid(data, k, clMethods = c("kmeans"), validation = "internal",

maxitems = nrow(data), metric = "correlation")

return(clusters(cor_kmeans, "kmeans")[[1]]$cluster)

}

# Correlation + HClust

cluster_function2 <- function(data, k) {

cor_hclust <- clValid(data, k, clMethods = c("hierarchical"), validation = "internal",

maxitems = nrow(data), metric = "correlation")

return(cutree(cor_hclust@clusterObjs$hierarchical, k))

}

# Gower + K-Medoids

cluster_function3 <- function(data, k) {

gower_dist <- daisy(data, metric = "gower")

pam_fit <- pam(gower_dist, diss = TRUE, k = k)

return(pam_fit$clustering)

}

# Gower + HClust

cluster_function4 <- function(data, k) {

gower_dist <- daisy(data, metric = "gower")

divisive_clust <- diana(as.matrix(gower_dist), diss = TRUE, keep.diss = TRUE)

return(cutree(divisive_clust, k = k))

}

# Number of clusters

k <- readline()

# Number of bootstrap samples

n_bootstrap <- readline()

# Number of rows to exclude from each bootstrap sample

n_exclude <- round(nrow(data) * 0.2) # Adjust this number based on your dataset size and requirements

# Function to calculate specificity and sensitivity

calculate_metrics <- function(true_labels, predicted_labels) {

confusion <- confusionMatrix(as.factor(predicted_labels), as.factor(true_labels))

sensitivity <- confusion$byClass["Sensitivity"]

specificity <- confusion$byClass["Specificity"]

return(c(Sensitivity = sensitivity, Specificity = specificity))

}

# Function to compare clusters using adjustedRandIndex

compare_clusters <- function(clusters1, clusters2) {

# Remove NAs for comparison

valid_indices <- which(!is.na(clusters1) & !is.na(clusters2))

if (length(valid_indices) == 0) return(NA)

return(adjustedRandIndex(clusters1[valid_indices], clusters2[valid_indices]))

}

# Function to check if cluster swapping is needed and perform the swap

adjust_clusters_if_needed <- function(true_labels, predicted_clusters) {

# Compute confusion matrix

conf_matrix <- table(TrueLabels = true_labels, PredictedClusters = predicted_clusters)

# Extract values from confusion matrix

true_1_pred_1 <- ifelse("1" %in% rownames(conf_matrix) & "1" %in% colnames(conf_matrix), conf_matrix["1", "1"], 0)

true_1_pred_2 <- ifelse("1" %in% rownames(conf_matrix) & "2" %in% colnames(conf_matrix), conf_matrix["1", "2"], 0)

true_2_pred_1 <- ifelse("2" %in% rownames(conf_matrix) & "1" %in% colnames(conf_matrix), conf_matrix["2", "1"], 0)

true_2_pred_2 <- ifelse("2" %in% rownames(conf_matrix) & "2" %in% colnames(conf_matrix), conf_matrix["2", "2"], 0)

# Compute proportions of patients assigned to the opposite cluster

prop_true_1_pred_2 <- true_1_pred_2 / (true_1_pred_1 + true_1_pred_2)

prop_true_2_pred_1 <- true_2_pred_1 / (true_2_pred_1 + true_2_pred_2)

# Check if swapping is needed based on the threshold (50%)

swap_needed <- (prop_true_1_pred_2 > 0.5) & (prop_true_2_pred_1 > 0.5)

# Perform swap if needed

if (swap_needed) {

# Determine which cluster should be swapped

swapped_clusters <- ifelse(predicted_clusters == 1, 2, 1)

} else {

swapped_clusters <- predicted_clusters

}

return(swapped_clusters)

}

# Existing percentage_matching function

percentage_matching <- function(vec1, vec2) {

if (length(vec1) != length(vec2)) {

stop("Vectors must be of the same length.")

}

valid_indices <- which(!is.na(vec1) & !is.na(vec2))

matches <- sum(vec1[valid_indices] == vec2[valid_indices])

percentage <- (matches / length(valid_indices)) * 100

return(percentage)

}

# Bootstrap_metrics function

bootstrap_metrics <- function(cluster_function, data, true_labels, k, n_bootstrap, n_exclude) {

# Store the metrics for each bootstrap sample

metrics_list <- matrix(NA, nrow = n_bootstrap, ncol = 2)

colnames(metrics_list) <- c("Sensitivity", "Specificity")

# Store the clustering results for each bootstrap sample

bootstrap_clusters <- matrix(NA, nrow = nrow(data), ncol = n_bootstrap)

# Perform bootstrapping

set.seed(42) # for reproducibility

for (i in 1:n_bootstrap) {

# Generate indices for rows to exclude

exclude_indices <- sample(1:nrow(data), n_exclude, replace = FALSE)

# Generate the bootstrap sample by excluding the selected indices

bootstrap_sample_indices <- setdiff(1:nrow(data), exclude_indices)

bootstrap_sample <- data[bootstrap_sample_indices, , drop = FALSE]

# Perform clustering on the bootstrap sample

clusters <- cluster_function(bootstrap_sample, k)

# Create a complete cluster assignment for the original data

full_clusters <- rep(NA, nrow(data))

full_clusters[bootstrap_sample_indices] <- clusters

# Adjust clusters if needed

adjusted_clusters <- adjust_clusters_if_needed(true_labels, full_clusters)

# Store the complete cluster assignment

bootstrap_clusters[, i] <- adjusted_clusters

# Calculate metrics only for non-NA cluster assignments

valid_indices <- !is.na(full_clusters)

if (sum(valid_indices) > 0) {

metrics <- calculate_metrics(true_labels[valid_indices], adjusted_clusters[valid_indices])

metrics_list[i, ] <- metrics

}

}

# Calculate average metrics

avg_metrics <- colMeans(metrics_list, na.rm = TRUE)

# Calculate stability (mean percentage matching) across bootstrap samples

similarity_matrix <- matrix(NA, nrow = n_bootstrap, ncol = n_bootstrap)

for (i in 1:n_bootstrap) {

for (j in 1:n_bootstrap) {

similarity_matrix[i, j] <- percentage_matching(bootstrap_clusters[, i], bootstrap_clusters[, j])

}

}

avg_stability <- mean(similarity_matrix[lower.tri(similarity_matrix, diag = FALSE)], na.rm = TRUE)

return(c(AverageSensitivity = avg_metrics["Sensitivity"], AverageSpecificity = avg_metrics["Specificity"], AverageStability = avg_stability))

}

# Example usage with one method

result <- bootstrap_metrics(cluster_function1, data, true_labels_numeric, k, n_bootstrap, n_exclude)

print(result)

# Define clustering methods

clustering_methods <- list(

"Correlation + K-Means" = cluster_function1,

"Correlation + HClust" = cluster_function2,

"Gower + K-Medoids" = cluster_function3,

"Gower + HClust" = cluster_function4

)

# Results list

results <- list()

# Calculate metrics and stability for each method

for (method_name in names(clustering_methods)) {

cat("Processing", method_name, "\n")

metrics <- bootstrap_metrics(clustering_methods[[method_name]], data, true_labels_numeric, k, n_bootstrap, n_exclude)

results[[method_name]] <- metrics

}

results_df <- do.call(rbind.data.frame, results)

rownames(results_df) <- names(clustering_methods)

colnames(results_df) <- c("Sensitivity", "Specificity", "Stability")

# Print results

print(results_df)

#### CLUSTALL METHOD ####

clustall <- function(data){

obj_noNA <- createClustAll(data = data, nImputation = NULL,

dataImputed = NULL)

obj_noNA1 <- runClustAll(Object = obj_noNA, threads = 2, simplify = FALSE)

res <- resStratification(Object = obj_noNA1, population = 0.05, stratification_similarity = 0.9,

all = FALSE)

return(obj_noNA1@summary_clusters[names(res[1])])

}

# Number of patients to exclude

n_exclude <- round(nrow(data) * 0.2)

# Data to assess

data <- data_use[, -1]

true_labels <- data_use[, 1] # True labels (diagnosis)

# Convert true_labels to numeric for comparison

true_labels_numeric <- as.numeric(as.factor(true_labels))

# Function to calculate specificity and sensitivity

calculate_metrics <- function(true_labels, predicted_labels) {

confusion <- confusionMatrix(as.factor(predicted_labels), as.factor(true_labels))

sensitivity <- confusion$byClass["Sensitivity"]

specificity <- confusion$byClass["Specificity"]

return(c(Sensitivity = sensitivity, Specificity = specificity))

}

# Function to compare clusters using adjustedRandIndex

compare_clusters <- function(clusters1, clusters2) {

# Remove NAs for comparison

valid_indices <- which(!is.na(clusters1) & !is.na(clusters2))

if (length(valid_indices) == 0) return(NA)

return(adjustedRandIndex(clusters1[valid_indices], clusters2[valid_indices]))

}

# Function to check if cluster swapping is needed and perform the swap

adjust_clusters_if_needed <- function(true_labels, predicted_clusters) {

# Compute confusion matrix

conf_matrix <- table(TrueLabels = true_labels, PredictedClusters = predicted_clusters)

# Extract values from confusion matrix

true_1_pred_1 <- ifelse("1" %in% rownames(conf_matrix) & "1" %in% colnames(conf_matrix), conf_matrix["1", "1"], 0)

true_1_pred_2 <- ifelse("1" %in% rownames(conf_matrix) & "2" %in% colnames(conf_matrix), conf_matrix["1", "2"], 0)

true_2_pred_1 <- ifelse("2" %in% rownames(conf_matrix) & "1" %in% colnames(conf_matrix), conf_matrix["2", "1"], 0)

true_2_pred_2 <- ifelse("2" %in% rownames(conf_matrix) & "2" %in% colnames(conf_matrix), conf_matrix["2", "2"], 0)

# Compute proportions of patients assigned to the opposite cluster

prop_true_1_pred_2 <- true_1_pred_2 / (true_1_pred_1 + true_1_pred_2)

prop_true_2_pred_1 <- true_2_pred_1 / (true_2_pred_1 + true_2_pred_2)

# Check if swapping is needed based on the threshold (50%)

swap_needed <- (prop_true_1_pred_2 > 0.5) & (prop_true_2_pred_1 > 0.5)

# Perform swap if needed

if (swap_needed) {

# Determine which cluster should be swapped

swapped_clusters <- ifelse(predicted_clusters == 1, 2, 1)

} else {

swapped_clusters <- predicted_clusters

}

return(swapped_clusters)

}

# Existing percentage_matching function

percentage_matching <- function(vec1, vec2) {

if (length(vec1) != length(vec2)) {

stop("Vectors must be of the same length.")

}

valid_indices <- which(!is.na(vec1) & !is.na(vec2))

matches <- sum(vec1[valid_indices] == vec2[valid_indices])

percentage <- (matches / length(valid_indices)) * 100

return(percentage)

}

# Number of bootstrap samples

n_bootstrap <- 100

bootstrap_metrics <- function(cluster_function, data, true_labels_numeric, n_bootstrap, n_exclude) {

# Store the metrics for each bootstrap sample

metrics_list <- matrix(NA, nrow = n_bootstrap, ncol = 2)

colnames(metrics_list) <- c("Sensitivity", "Specificity")

# Store the clustering results for each bootstrap sample

bootstrap_clusters <- matrix(NA, nrow = nrow(data), ncol = n_bootstrap)

set.seed(42) # for reproducibility

for (i in 1:n_bootstrap) {

cat("Bootsrapping iteration:", i, "\n")

# Generate indices for rows to exclude

exclude_indices <- sample(1:nrow(data), n_exclude, replace = FALSE)

# Generate the bootstrap sample by excluding the selected indices

bootstrap_sample_indices <- setdiff(1:nrow(data), exclude_indices)

# Generate the bootstrap sample by excluding the selected indices, we don't consider the missing values here

data_bootstrap <- data[bootstrap_sample_indices,]

clusters <- unname(unlist(clustall(data_bootstrap)))

# Create a complete cluster assignment for the original data

full_clusters <- rep(NA, nrow(data))

full_clusters[bootstrap_sample_indices] <- clusters

# Adjust clusters if needed

adjusted_clusters <- adjust_clusters_if_needed(true_labels_numeric, full_clusters)

# Store the complete cluster assignment

bootstrap_clusters[, i] <- adjusted_clusters

# Calculate metrics only for non-NA cluster assignments

valid_indices <- !is.na(full_clusters)

if (sum(valid_indices) > 0) {

metrics <- calculate_metrics(true_labels_numeric[valid_indices], adjusted_clusters[valid_indices])

metrics_list[i, ] <- metrics

}

}

# Calculate average metrics

avg_metrics <- colMeans(metrics_list, na.rm = TRUE)

# Calculate stability (mean percentage matching) across bootstrap samples

similarity_matrix <- matrix(NA, nrow = n_bootstrap, ncol = n_bootstrap)

for (i in 1:n_bootstrap) {

for (j in 1:n_bootstrap) {

similarity_matrix[i, j] <- percentage_matching(bootstrap_clusters[, i], bootstrap_clusters[, j])

}

}

avg_stability <- mean(similarity_matrix[lower.tri(similarity_matrix, diag = FALSE)], na.rm = TRUE)

return(c(AverageSensitivity = avg_metrics["Sensitivity"], AverageSpecificity = avg_metrics["Specificity"], AverageStability = avg_stability))

}

# Run and save results

clustall_results <- bootstrap_metrics(clustall, data, true_labels_numeric, n_bootstrap, n_exclude)

# Print results

print(clustall_results)

**ClustAll computational efficiency assessment**

# Load required packages

library(ClustAll)

library(tidyverse)

library(parallel)

library(microbenchmark)

library(ggplot2)

library(caret)

# Set random seed for reproducibility

set.seed(123)

###########################################

# Load Linear ClustALL Implementation

###########################################

# Note: You need to modify the path to where you downloaded the Linear ClustALL code

# from https://github.com/TranslationalBioinformaticsUnit/ClustALL_AD/tree/main/R_code

source("path/to/00_Functions.R")

###########################################

# Part A: Linear vs S4 Performance Comparison

###########################################

# Function to create datasets with different number of variables

create_subset_data <- function(data, n_vars) {

# Keep Diagnosis and select n_vars from remaining columns

subset_cols <- c("Diagnosis",

sample(setdiff(names(data), c("Diagnosis")), n_vars))

return(data[, subset_cols])

}

# Load both complete and missing datasets

data("BreastCancerWisconsin", package = "ClustAll")

data("BreastCancerWisconsinMISSING", package = "ClustAll")

data_complete <- subset(wdbc, select = -ID)

data_missing <- wdbcNA

# Variable sizes to test as specified in the manuscript

var_sizes <- c(15, 20, 25, 30)

# Initialize results dataframe

results_comparison <- data.frame(

n_vars = rep(var_sizes, each = 4),

method = rep(rep(c("Linear_ClustALL", "S4 ClustAll"), each = 2), times = length(var_sizes)),

imputation = rep(c("No Imputation", "10 Imputations"), times = length(var_sizes) * 2),

runtime = NA

)

# Function to run Linear ClustALL

run_linear_clustall <- function(obj, n_imp = NULL) {

start_time <- Sys.time()

if(is.null(n_imp)) {

# Run Linear ClustALL without imputation

ClustALL_linear_noImp(obj@data)

} else {

# Run Linear ClustALL with imputation

ClustALL_linear_withImp(obj@dataImputed, nimp = 10)

}

end_time <- Sys.time()

return(as.numeric(difftime(end_time, start_time, units = "secs")))

}

# Function to run benchmarking (10 iterations)

run_benchmark <- function(data, method, n_imp = NULL, n_iterations = 10) {

times <- numeric(n_iterations)

for(i in 1:n_iterations) {

obj <- createClustAll(data = data,

nImputation = n_imp,

dataImputed = NULL,

colValidation = "Diagnosis")

if(method == "S4 ClustAll") {

start_time <- Sys.time()

result <- runClustAll(Object = obj, threads = 1, simplify = FALSE)

end_time <- Sys.time()

times[i] <- as.numeric(difftime(end_time, start_time, units = "secs"))

} else {

times[i] <- run_linear_clustall(obj, n_imp)

}

}

return(mean(times))

}

# Run benchmarks for each combination

for(i in 1:nrow(results_comparison)) {

# Choose appropriate dataset based on imputation setting

if(results_comparison$imputation[i] == "10 Imputations") {

current_data <- create_subset_data(data_missing, results_comparison$n_vars[i])

n_imp <- 10

} else {

current_data <- create_subset_data(data_complete, results_comparison$n_vars[i])

n_imp <- NULL

}

cat("Running benchmark for", results_comparison$n_vars[i], "variables,",

results_comparison$method[i], "with", results_comparison$imputation[i], "\n")

results_comparison$runtime[i] <- run_benchmark(

current_data,

method = results_comparison$method[i],

n_imp = n_imp

)

}

###########################################

# Part B: Parallel Computing Performance

###########################################

# Core counts as specified in manuscript

core_counts <- c(1, 2, 4, 8, 16, 32, 64)

imp_scenarios <- c(0, 10, 100) # No imputation, 10 imputations, 100 imputations

# Initialize results dataframe for parallel benchmarking

results_parallel <- data.frame(

cores = rep(core_counts, each = length(imp_scenarios)),

imputations = rep(imp_scenarios, times = length(core_counts)),

runtime = NA

)

# Function to run parallel benchmarking (10 iterations)

run_parallel_benchmark <- function(data, n_cores, n_imp = NULL, n_iterations = 10) {

times <- numeric(n_iterations)

for(i in 1:n_iterations) {

obj <- createClustAll(data = data,

nImputation = n_imp,

dataImputed = NULL,

colValidation = "Diagnosis")

start_time <- Sys.time()

result <- runClustAll(Object = obj, threads = n_cores, simplify = FALSE)

end_time <- Sys.time()

times[i] <- as.numeric(difftime(end_time, start_time, units = "secs"))

}

return(mean(times))

}

# Run parallel benchmarks

for(i in 1:nrow(results_parallel)) {

# Choose appropriate dataset based on imputation setting

if(results_parallel$imputations[i] > 0) {

current_data <- data_missing

n_imp <- results_parallel$imputations[i]

} else {

current_data <- data_complete

n_imp <- NULL

}

cat("Running parallel benchmark with", results_parallel$cores[i], "cores and",

results_parallel$imputations[i], "imputations\n")

results_parallel$runtime[i] <- run_parallel_benchmark(

current_data,

n_cores = results_parallel$cores[i],

n_imp = n_imp

)

}

#----------------------------------------------------------

## Linear ClustALL Functions

#----------------------------------------------------------

# Adapted function from the following script:

# https://github.com/TranslationalBioinformaticsUnit/ClustALL_AD/blob/main/R_code/01a_ClustALL_noImputation.R

# Run Linear ClustALL Algorithm without Imputation

ClustALL_linear_noImp <- function(data_use, population_threshold = 0.05) {

# Input validation

if (!is.data.frame(data_use)) {

stop("Input data must be a data frame")

}

if (any(is.na(data_use))) {

stop("Input data contains missing values. Use the imputation version of ClustALL instead.")

}

# Define number of variables

nvariables <- ncol(data_use)

# Categorical variables are converted to binary with one-hot encoding

tobin <- data_use[, sapply(data_use, is.factor)]

if(ncol(tobin) > 0) {

dummy <- dummyVars(" ~ .", data=tobin)

bin <- data.frame(predict(dummy, newdata=tobin))

names(bin) <- names(tobin)

data_use <- data_use[, !(names(data_use) %in% names(tobin))]

data_use <- cbind(data_use, bin)

}

# Convert to numeric

data_use <- data.frame(lapply(data_use, function(x) {

if(!is.numeric(x)) as.numeric(as.character(x)) else x

}))

# Convert to numeric

data_use[,1:nvariables] <- data.frame(apply(data_use[,1:nvariables], 2, as.numeric))

# Initialize matrices

summary_clusters_a <- matrix(0,nvariables,nimp)

summary_clusters_b <- matrix(0,nvariables,nimp)

summary_clusters_c <- matrix(0,nvariables,nimp)

summary_clusters_d <- matrix(0,nvariables,nimp)

summary_matrices_a <- vector("list",nvariables)

summary_matrices_b <- vector("list",nvariables)

summary_matrices_c <- vector("list",nvariables)

summary_matrices_d <- vector("list",nvariables)

# Initialize matrix templates

for(i in 1:nvariables) {

m_template <- matrix(0, nrow(data_use), nrow(data_use))

rownames(m_template) <- colnames(m_template) <- rownames(data_use)

summary_matrices_a[[i]] <- summary_matrices_b[[i]] <- summary_matrices_c[[i]] <- summary_matrices_d[[i]] <- m_template

}

# Data Complexity Reduction

cor_est_var <- cor(data_use, method="spearman")

variables_clust <- hclust(as.dist(cor_est_var))

possible_heights <- variables_clust$height

# Process each depth

for(heights_cut in (length(possible_heights)-(ncol(data_use)-2)):(length(possible_heights)-1)) {

# Cut tree and get groups

treego <- cutree(variables_clust, h=possible_heights[heights_cut])

groups_go <- unique(treego)

# Process first group

variables_use <- names(treego[treego==groups_go[1]])

data_PCA <- if(length(variables_use)==1) {

data_use[,variables_use]

} else {

generatePCA_derived(data=data_use[,variables_use], variability=50, maxvar=3)

}

# Process additional groups

if(length(groups_go) > 1) {

for(h in 2:length(groups_go)) {

variables_use <- names(treego[treego==groups_go[h]])

if(length(variables_use)==1) {

data_PCA <- cbind(data_PCA, data_use[,variables_use])

} else {

data_PCA <- cbind(data_PCA, generatePCA_derived(data=data_use[,variables_use],

variability=50, maxvar=3))

}

}

}

# Scale PCA data

data_PCA_scaled <- apply(data_PCA, 2, scale)

rownames(data_PCA_scaled) <- 1:nrow(data_PCA_scaled)

# Process Correlation + K-means

data_PCA.clValid_internal_kmeans <- clValid(data_PCA_scaled, 2:6,

clMethods=c("kmeans"),

validation="internal",

maxitems=nrow(data_PCA_scaled),

metric="correlation")

oS_kmeans <- as.numeric(mlv(optimalScores(data_PCA.clValid_internal_kmeans)[,3],

method = "mfv"))

if(length(oS_kmeans) > 1) oS_kmeans <- median(oS_kmeans)

kmeans_res <- clusters(data_PCA.clValid_internal_kmeans, "kmeans")

kmeans_res_c <- kmeans_res[as.character(oS_kmeans)][[1]]$cluster

for(t in 1:as.numeric(oS_kmeans)) {

induse <- as.numeric(names(kmeans_res_c[kmeans_res_c==t]))

summary_matrices_a[[heights_cut]][induse, induse] <-

summary_matrices_a[[heights_cut]][induse, induse] + 1

}

summary_clusters_a[heights_cut, 1] <- oS_kmeans

# Process Correlation + H-Clust

data_PCA.clValid_internal_hclust <- clValid(data_PCA_scaled, 2:6,

clMethods=c("hierarchical"),

validation="internal",

maxitems=nrow(data_PCA_scaled),

metric="correlation")

oS_hclust <- as.numeric(mlv(optimalScores(data_PCA.clValid_internal_hclust)[,3],

method = "mfv"))

if(length(oS_hclust) > 1) oS_hclust <- median(oS_hclust)

hclust_res <- clusters(data_PCA.clValid_internal_hclust, "hierarchical")

hclust_res_c <- cutree(hclust_res, k=oS_hclust)

for(t in 1:oS_hclust) {

induse <- as.numeric(names(hclust_res_c[hclust_res_c==t]))

summary_matrices_b[[heights_cut]][induse, induse] <-

summary_matrices_b[[heights_cut]][induse, induse] + 1

}

summary_clusters_b[heights_cut, 1] <- oS_hclust

# Process Gower distances

PCA_gower <- as.data.frame(data_PCA)

colnames(PCA_gower) <- c(1:dim(PCA_gower)[2])

binary <- which(apply(PCA_gower,2,function(x) {all(x %in% 0:1)}))

if(length(binary)==1) {

PCA_gower[,binary] <- factor(PCA_gower[,binary])

} else if(length(binary) > 1) {

PCA_gower[,binary] <- data.frame(apply(PCA_gower[,binary], 2, as.logical))

}

PCA_gower <- PCA_gower %>% mutate_if(is.character, as.factor)

gower_dist <- daisy(PCA_gower, metric="gower")

# Process Gower + K-Medoids

summary_clusters_c[heights_cut, 1] <- cstats.table_PAM(gower_dist, 6)

pam_res <- pam(gower_dist, k=summary_clusters_c[heights_cut, 1])

pam_res_c <- pam_res$clustering

for(t in 1:summary_clusters_c[heights_cut, 1]) {

induse <- as.numeric(names(pam_res_c[pam_res_c==t]))

summary_matrices_c[[heights_cut]][induse, induse] <-

summary_matrices_c[[heights_cut]][induse, induse] + 1

}

# Process Gower + H-Clust

divisive.clust <- diana(as.matrix(gower_dist), diss = TRUE, keep.diss = TRUE)

summary_clusters_d[heights_cut, 1] <- cstats.table_hclust(gower_dist, divisive.clust, 6)

hclustgow_res_c <- cutree(divisive.clust, k=summary_clusters_d[heights_cut, 1])

names(hclustgow_res_c) <- 1:dim(data_use)[1]

for(t in 1:summary_clusters_d[heights_cut, 1]) {

induse <- as.numeric(names(hclustgow_res_c[hclustgow_res_c==t]))

summary_matrices_d[[heights_cut]][induse, induse] <-

summary_matrices_d[[heights_cut]][induse, induse] + 1

}

}

# Process results

nclust_a <- as.data.frame(summary_clusters_a[which(apply(summary_clusters_a, 1, sum)>0),])

nclust_b <- as.data.frame(summary_clusters_b[which(apply(summary_clusters_b, 1, sum)>0),])

nclust_c <- as.data.frame(summary_clusters_c[which(apply(summary_clusters_c, 1, sum)>0),])

nclust_d <- as.data.frame(summary_clusters_d[which(apply(summary_clusters_d, 1, sum)>0),])

summary_n_clust <- c(apply(nclust_a, 1, function(x) median(x, na.rm=T)),

apply(nclust_b, 1, function(x) median(x, na.rm=T)),

apply(nclust_c, 1, function(x) median(x, na.rm=T)),

apply(nclust_d, 1, function(x) median(x, na.rm=T)))

# Name matrices

names(summary_matrices_a) <- paste("cuts_a_", 1:length(summary_matrices_a), sep="")

names(summary_matrices_b) <- paste("cuts_b_", 1:length(summary_matrices_b), sep="")

names(summary_matrices_c) <- paste("cuts_c_", 1:length(summary_matrices_c), sep="")

names(summary_matrices_d) <- paste("cuts_d_", 1:length(summary_matrices_d), sep="")

summary_matrices_MEASURES <- c(summary_matrices_a, summary_matrices_b,

summary_matrices_c, summary_matrices_d)

# Remove empty matrices

tmp <- lapply(summary_matrices_MEASURES, function(x) as.matrix(x[]))

if(length(which(lapply(tmp, sum)==0)) > 0) {

summary_matrices_MEASURES <- summary_matrices_MEASURES[-which(lapply(summary_matrices_MEASURES,

function(x) sum(x[]))==0)]

}

# Create summary clusters

summary_clusters <- vector("list", length(summary_matrices_MEASURES))

for(i in 1:length(summary_clusters)) {

hclustgo <- hclust(1-as.dist(summary_matrices_MEASURES[[i]][]))

summary_clusters[[i]] <- cutree(hclustgo, k=summary_n_clust[i])

}

names(summary_clusters) <- names(summary_matrices_MEASURES)

# Calculate Jaccard Distance

JACCARD_DISTANCE <- matrix(NA, length(summary_matrices_MEASURES), length(summary_matrices_MEASURES))

rownames(JACCARD_DISTANCE) <- colnames(JACCARD_DISTANCE) <- names(summary_matrices_MEASURES)

for(i in 1:nrow(JACCARD_DISTANCE)) {

for(j in 1:nrow(JACCARD_DISTANCE)) {

JACCARD_DISTANCE[i,j] <- cluster_similarity(summary_clusters[[i]],

summary_clusters[[j]],

similarity="jaccard",

method="independence")

}

}

# Calculate stability

summary_matrices_STABILITY <- matrix(NA, length(summary_matrices_MEASURES), 3)

for(i in 1:length(summary_matrices_MEASURES)){

invisible(capture.output(

r1 <- clusterboot(data=as.dist(1000-summary_matrices_MEASURES[[i]]),

B=100, distances=TRUE, bootmethod="boot",

bscompare=TRUE, multipleboot=FALSE,

jittertuning=0.05, noisetuning=c(0.05,4),

subtuning=floor(nrow(data_use)/2),

clustermethod=disthclustCBI,noisemethod=FALSE,

count=TRUE, showplots=FALSE,

dissolution=0.5, recover=0.75,

method="complete",k=2)

))

summary_matrices_STABILITY[i,1:2] <- as.numeric(r1$bootmean[1:2])

summary_matrices_STABILITY[i,3] <- mean(summary_matrices_STABILITY[i,1:2])

}

# Filter by stability

quantileuse <- 0.85

qgo <- quantile(summary_matrices_STABILITY[,3], quantileuse)

JACCARD_DISTANCE_F <- JACCARD_DISTANCE[summary_matrices_STABILITY[,3] >= qgo,

summary_matrices_STABILITY[,3] >= qgo]

return(JACCARD_DISTANCE_F)

}

# Adapted function from the following script:

# https://github.com/TranslationalBioinformaticsUnit/ClustALL_AD/blob/main/R_code/01b_ClustALL_Imputation.R

ClustALL_linear_withImp <- function(data, nimp = 10) {

imp <- data

data_use <- data$data

nvariables <- ncol(data_use)

# Create empty matrices for clustering

summary_clusters_a <- matrix(0, nvariables, nimp)

summary_clusters_b <- matrix(0, nvariables, nimp)

summary_clusters_c <- matrix(0, nvariables, nimp)

summary_clusters_d <- matrix(0, nvariables, nimp)

summary_matrices_a <- vector("list", nvariables)

summary_matrices_b <- vector("list", nvariables)

summary_matrices_c <- vector("list", nvariables)

summary_matrices_d <- vector("list", nvariables)

# Initialize matrices with proper row and column names

for(i in 1:nvariables) {

m_template <- matrix(0, nrow(data_use), nrow(data_use))

rownames(m_template) <- colnames(m_template) <- rownames(data_use)

summary_matrices_a[[i]] <- m_template

summary_matrices_b[[i]] <- m_template

summary_matrices_c[[i]] <- m_template

summary_matrices_d[[i]] <- m_template

}

# Process each imputation

for(impgo in 1:nimp) {

# Get completed data for this imputation

data_use <- mice::complete(imp, impgo)

# Calculate correlations and cluster variables

cor_est_var <- cor(data_use, method="spearman")

variables_clust <- hclust(as.dist(cor_est_var))

possible_heights <- variables_clust$height

# Process each depth

for(heights_cut in (length(possible_heights)-(ncol(data_use)-2)):(length(possible_heights)-1)) {

treego <- cutree(variables_clust, h=possible_heights[heights_cut])

groups_go <- unique(treego)

# Process first group

variables_use <- names(treego[treego==groups_go[1]])

if(length(variables_use)==1) {

data_PCA <- data_use[,variables_use, drop=FALSE]

} else {

data_PCA <- generatePCA_derived(data=data_use[,variables_use], variability=50, maxvar=3)

}

# Process additional groups

if(length(groups_go) > 1) {

for(h in 2:length(groups_go)) {

variables_use <- names(treego[treego==groups_go[h]])

if(length(variables_use)==1) {

data_PCA <- cbind(data_PCA, data_use[,variables_use])

} else {

data_PCA <- cbind(data_PCA, generatePCA_derived(data=data_use[,variables_use],

variability=50, maxvar=3))

}

}

}

# Scale PCA data

data_PCA_scaled <- apply(data_PCA, 2, scale)

rownames(data_PCA_scaled) <- 1:nrow(data_PCA_scaled)

# Process Correlation + K-means

data_PCA.clValid_internal_kmeans <- clValid(data_PCA_scaled, 2:6,

clMethods=c("kmeans"),

validation="internal",

maxitems=nrow(data_PCA_scaled),

metric="correlation")

oS_kmeans <- as.numeric(mlv(optimalScores(data_PCA.clValid_internal_kmeans)[,3],

method = "mfv"))

if(length(oS_kmeans) > 1) oS_kmeans <- median(oS_kmeans)

kmeans_res <- clusters(data_PCA.clValid_internal_kmeans, "kmeans")

kmeans_res_c <- kmeans_res[as.character(oS_kmeans)][[1]]$cluster

for(t in 1:as.numeric(oS_kmeans)) {

induse <- as.numeric(names(kmeans_res_c[kmeans_res_c==t]))

summary_matrices_a[[heights_cut]][induse, induse] <-

summary_matrices_a[[heights_cut]][induse, induse] + 1

}

summary_clusters_a[heights_cut, impgo] <- oS_kmeans

# Process Correlation + H-Clust

data_PCA.clValid_internal_hclust <- clValid(data_PCA_scaled, 2:6,

clMethods=c("hierarchical"),

validation="internal",

maxitems=nrow(data_PCA_scaled),

metric="correlation")

oS_hclust <- as.numeric(mlv(optimalScores(data_PCA.clValid_internal_hclust)[,3],

method = "mfv"))

if(length(oS_hclust) > 1) oS_hclust <- median(oS_hclust)

hclust_res <- clusters(data_PCA.clValid_internal_hclust, "hierarchical")

hclust_res_c <- cutree(hclust_res, k=oS_hclust)

for(t in 1:oS_hclust) {

induse <- as.numeric(names(hclust_res_c[hclust_res_c==t]))

summary_matrices_b[[heights_cut]][induse, induse] <-

summary_matrices_b[[heights_cut]][induse, induse] + 1

}

summary_clusters_b[heights_cut, impgo] <- oS_hclust

# Process Gower distance

PCA_gower <- as.data.frame(data_PCA)

colnames(PCA_gower) <- paste0("V", 1:ncol(PCA_gower))

binary <- which(apply(PCA_gower, 2, function(x) all(x %in% 0:1)))

if(length(binary) > 0) {

if(length(binary)==1) {

PCA_gower[,binary] <- factor(PCA_gower[,binary])

} else {

PCA_gower[,binary] <- data.frame(apply(PCA_gower[,binary], 2, as.logical))

}

}

PCA_gower <- PCA_gower %>% mutate_if(is.character, as.factor)

gower_dist <- daisy(PCA_gower, metric="gower")

# Process Gower + K-Medoids (PAM)

oS_pam <- cstats.table_PAM(gower_dist, 6)

pam_res <- pam(gower_dist, k=oS_pam)

pam_res_c <- pam_res$clustering

for(t in 1:oS_pam) {

induse <- which(pam_res_c == t)

summary_matrices_c[[heights_cut]][induse, induse] <-

summary_matrices_c[[heights_cut]][induse, induse] + 1

}

summary_clusters_c[heights_cut, impgo] <- oS_pam

# Process Gower + H-Clust

divisive.clust <- diana(as.matrix(gower_dist), diss=TRUE, keep.diss=TRUE)

oS_diana <- cstats.table_hclust(gower_dist, divisive.clust, 6)

diana_res_c <- cutree(divisive.clust, k=oS_diana)

for(t in 1:oS_diana) {

induse <- which(diana_res_c == t)

summary_matrices_d[[heights_cut]][induse, induse] <-

summary_matrices_d[[heights_cut]][induse, induse] + 1

}

summary_clusters_d[heights_cut, impgo] <- oS_diana

}

}

# Process results

nclust_a <- as.data.frame(summary_clusters_a[which(apply(summary_clusters_a, 1, sum)>0),])

nclust_b <- as.data.frame(summary_clusters_b[which(apply(summary_clusters_b, 1, sum)>0),])

nclust_c <- as.data.frame(summary_clusters_c[which(apply(summary_clusters_c, 1, sum)>0),])

nclust_d <- as.data.frame(summary_clusters_d[which(apply(summary_clusters_d, 1, sum)>0),])

summary_n_clust <- c(apply(nclust_a, 1, function(x) median(x, na.rm=T)),

apply(nclust_b, 1, function(x) median(x, na.rm=T)),

apply(nclust_c, 1, function(x) median(x, na.rm=T)),

apply(nclust_d, 1, function(x) median(x, na.rm=T)))

# Rename matrices

names(summary_matrices_a) <- paste("cuts_a_", 1:length(summary_matrices_a), sep="")

names(summary_matrices_b) <- paste("cuts_b_", 1:length(summary_matrices_b), sep="")

names(summary_matrices_c) <- paste("cuts_c_", 1:length(summary_matrices_c), sep="")

names(summary_matrices_d) <- paste("cuts_d_", 1:length(summary_matrices_d), sep="")

summary_matrices_MEASURES <- c(summary_matrices_a, summary_matrices_b,

summary_matrices_c, summary_matrices_d)

# Remove empty matrices

tmp <- lapply(summary_matrices_MEASURES, function(x) as.matrix(x[]))

if(length(which(lapply(tmp, sum)==0)) > 0) {

summary_matrices_MEASURES <- summary_matrices_MEASURES[-which(lapply(tmp, sum)==0)]

}

# Create summary clusters

summary_clusters <- vector("list", length(summary_matrices_MEASURES))

for(i in 1:length(summary_clusters)) {

hclustgo <- hclust(1-as.dist(summary_matrices_MEASURES[[i]][]))

summary_clusters[[i]] <- cutree(hclustgo, k=summary_n_clust[i])

}

names(summary_clusters) <- names(summary_matrices_MEASURES)

# Calculate Jaccard Distance

JACCARD_DISTANCE <- matrix(NA, length(summary_matrices_MEASURES), length(summary_matrices_MEASURES))

rownames(JACCARD_DISTANCE) <- colnames(JACCARD_DISTANCE) <- names(summary_matrices_MEASURES)

for(i in 1:nrow(JACCARD_DISTANCE)) {

for(j in 1:nrow(JACCARD_DISTANCE)) {

JACCARD_DISTANCE[i,j] <- cluster_similarity(summary_clusters[[i]],

summary_clusters[[j]],

similarity="jaccard",

method="independence")

}

}

# Calculate stability

summary_matrices_STABILITY <- matrix(NA, length(summary_matrices_MEASURES), 3)

for(i in 1:length(summary_matrices_MEASURES)) {

invisible(capture.output(

r1 <- clusterboot(data=as.dist(1000-summary_matrices_MEASURES[[i]]),

B=100, distances=TRUE, bootmethod="boot",

bscompare=TRUE, multipleboot=FALSE,

jittertuning=0.05, noisetuning=c(0.05,4),

subtuning=floor(nrow(data_use)/2),

clustermethod=disthclustCBI, noisemethod=FALSE,

count=TRUE, showplots=FALSE,

dissolution=0.5, recover=0.75,

method="complete", k=2)

))

summary_matrices_STABILITY[i,1:2] <- as.numeric(r1$bootmean[1:2])

summary_matrices_STABILITY[i,3] <- mean(summary_matrices_STABILITY[i,1:2])

}

# Filter by stability

quantileuse <- 0.85

qgo <- quantile(summary_matrices_STABILITY[,3], quantileuse)

JACCARD_DISTANCE_F <- JACCARD_DISTANCE[summary_matrices_STABILITY[,3] >= qgo,

summary_matrices_STABILITY[,3] >= qgo]

return(JACCARD_DISTANCE_F)

}
